# Associations between serological evidence of SARS-CoV-2 infection and longitudinal pulmonary outcomes among people with HIV: analysis of the MACS/WIHS combined cohort study (MWCCS)

**DOI:** 10.1186/s12931-026-03542-4

**Published:** 2026-02-05

**Authors:** Catie A Wiener, Andrew Edmonds, Beverly E Sha, Valentina Stosor, Igor Z Barjaktarevic, Meredith C McCormack, Jodie A Dionne, Maria L Alcaide, Sushma K Cribbs, Stephen J Gange, Deepa G Lazarous, Robert F Foronjy, Divya B Reddy, Laurence Huang, Ken M Kunisaki, Alison Morris, Alicia E Diggs, Catalina Ramirez, Stephen R Cole, Lakshmanane Premkumar, Michelle A Floris-Moore, M. Bradley Drummond

**Affiliations:** 1 University of North Carolina at Chapel Hill, Chapel Hill, NC USA; 2 Rush University Medical Center, Chicago, IL USA; 3 Northwestern University Feinberg School of Medicine, Chicago, IL USA; 4 University of California at Los Angeles, Los Angeles, CA USA; 5 Johns Hopkins University, Baltimore, MD USA; 6 University of Alabama at Birmingham, Birmingham, AL USA; 7 University of Miami, Miami, FL USA; 8 Emory University, Atlanta, GA USA; 9 Georgetown University, Washington, D.C., USA; 10 SUNY Downstate Health Sciences University, Brooklyn, NY USA; 11 Albert Einstein College of Medicine, Bronx, NY USA; 12 University of California at San Francisco, San Franciso, CA USA; 13 Boise Veterans Affairs (VA) Medical Center, Boise, ID USA; 14 University of Pittsburgh, Pittsburgh, PA USA

**Keywords:** Pulmonary function test, SARS-CoV-2 infection, Human immunodeficiency virus

## Abstract

**Background:**

People with HIV (PWH) are at increased risk for chronic lung disease and may be more susceptible to pulmonary complications following severe acute respiratory syndrome coronavirus 2 (SARS-CoV-2) infection. It remains unclear whether HIV infection modifies long-term pulmonary outcomes after coronavirus disease 2019 (COVID-19). This study assessed longitudinal changes in pulmonary function and respiratory symptoms among PWH and people without HIV (PWoH) with serologically-confirmed SARS-CoV-2 infection.

**Methods:**

We analyzed data from the Multicenter AIDS Cohort Study (MACS)/Women’s Interagency HIV Study (WIHS) Combined Cohort Study (MWCCS), a prospective US cohort of PWH and PWoH. Participants with serologic evidence of past SARS-CoV-2 infection and acceptable pre- and post-SARS-CoV-2 pulmonary function testing (PFT) including spirometry and diffusing capacity for carbon monoxide (DLCO) were included. Annualized changes in post-bronchodilator (BD) forced expiratory volume in 1 second (FEV_1_), forced vital capacity (FVC), and DLCO were compared by HIV serostatus, stratified by sex. Respiratory symptoms were assessed using the St. George’s Respiratory Questionnaire (SGRQ). Linear regression estimated differences in pulmonary function change by HIV serostatus, stratifying by relevant covariates.

**Results:**

Among 778 participants (204 men; 574 women) exposed to SARS-CoV-2 and who underwent PFTs before and after infection, 66% were PWH. Men with HIV (MWH) had a mean annualized FEV_1_ decline of -44.3 mL/year versus -33.8 mL/year in men without HIV (MWoH) (mean difference -10.5 mL/year; 95% CI: -30.7, 9.7). Among women, FEV_1_ declined -19.8 mL/year in PWH vs. -14.8 mL/year in PWoH (mean difference -5.0 mL/year; 95% CI: -18.2, 8.2). Changes in FVC and DLCO were similar across HIV serostatus groups. No consistent differences in respiratory symptom changes were observed between PWH and PWoH. Subgroup analyses did not reveal any HIV-associated differential changes.

**Conclusions:**

Among individuals with serologic evidence of SARS-CoV-2 infection, HIV serostatus was not associated with greater declines in pulmonary function or worsening of respiratory symptoms. Our findings suggest that having HIV alone may not increase the risk for pulmonary impairments following a SARS-CoV-2 infection. Further research is needed to understand how uncontrolled HIV infection and severity of SARS-CoV-2 infection may increase risks of adverse pulmonary outcomes following a SARS-CoV-2 infection.

**Supplementary Information:**

The online version contains supplementary material available at 10.1186/s12931-026-03542-4.

## Introduction

Severe acute respiratory syndrome coronavirus 2 (SARS-CoV-2) emerged in late 2019 and became a severe global pandemic affecting over 178 countries [1, 2]. SARS-CoV-2 primarily impacts the respiratory system, with manifestations ranging from asymptomatic infection to severe pneumonia and subsequent acute respiratory distress syndrome [3]. Following resolution of acute infection, individuals can experience persistent pulmonary complications regardless of initial disease severity [4]. Pulmonary impairments following SARS-CoV-2 infection have been documented in individuals without underlying lung conditions, and include abnormalities such as reduced forced expiratory volume in 1 s (FEV_1_), forced vital capacity (FVC), and diffusing capacity for carbon monoxide (DLCO) [5–7].

Individuals living with chronic medical conditions, including those living with HIV, have been identified as disproportionately impacted by SARS-CoV-2 infection [8–10]. People with HIV (PWH) are also susceptible to increased risks of chronic lung impairments unrelated to SARS-CoV-2 infection [11, 12]. Despite effective antiretroviral therapy (ART), PWH may experience lung immune dysregulation [13] and increased susceptibility to respiratory infections. Other mechanisms contributing to adverse pulmonary outcomes include HIV-related structural and immunological changes within the lung, alterations in the lung microbiological milieu, and differential environmental exposures [14]. Chronic lung impairments among PWH manifest as mild abnormalities in pulmonary function testing (PFT) or clinically overt airway diseases (e.g., chronic obstructive pulmonary disease [COPD] and asthma), emphysema, interstitial lung diseases, and pulmonary hypertension [15–19]. PWH experience declines in FEV_1_ and FVC ranging from 25 to 62 ml/year and 9 to 67 ml/year respectively, depending upon cohort composition and follow-up duration. HIV infection is associated with excess loss of 10–17 ml/year in FEV_1_, 13 ml/year in FVC, and 0.08 ml/min/mm Hg/year in DLCO, values comparable to the impact of active smoking in the general population [19]. In addition to the pre-existing pulmonary vulnerabilities seen in PWH, this population has biological susceptibilities to adverse outcomes after SARS-CoV-2 infection. PWH demonstrate prolonged SARS-CoV-2 viral shedding, potentially leading to prolonged pulmonary inflammation and tissue damage [20]. Chronic HIV infection is associated with persistent interferon signaling, a process associated with lack of protection against SARS-CoV-2 infection in gut epithelial cells, with similar potential compromised antiviral responses in other epithelial tissues such as the lungs [21]. Finally, reactivation of HIV provirus during SARS-CoV-2 infection has been hypothesized to result in endothelial dysfunction and apoptosis secondary to aberrant HIV protein expression, leading to pulmonary vascular remodeling and diffusion impairments [22]. Impairments in specific pulmonary measures could inform potential causal pathways, with FEV_1_ measurements reflecting aberrant airways inflammation, FVC measurements reflecting processes impacting interstitial fibrosis, and DLCO measurements capturing pulmonary vascular pathology. Given the respiratory tropism of SARS-CoV-2 and the risk of lung diseases in PWH, improved understanding of how these overlapping conditions impact longitudinal lung health among PWH is critical.

The Multicenter AIDS Cohort Study (MACS) [23] and Women’s Interagency HIV Study (WIHS) [24], now combined into the MACS/WIHS Combined Cohort Study (MWCCS) [25] are prospective observational cohorts of PWH and people without HIV (PWoH) at increased vulnerability for HIV in the United States. The cohorts conducted repeated PFTs, respiratory symptom assessments, and serological testing for evidence of past SARS-CoV-2 infection. These data provide a unique opportunity to determine whether HIV infection is associated with differential changes in these measures among individuals infected with SARS-CoV-2.

## Methods

In this analysis, we described changes in FEV_1_, FVC, and DLCO as well as respiratory symptoms among MWCCS participants with serological evidence of past SARS-CoV-2 infection. We hypothesized that PWH would have more substantial pulmonary function decline and worsening respiratory symptoms following a SARS-CoV-2 infection compared to PWoH.

### Study population

Prior to cohort merging, people enrolled in the MACS and the WIHS attended study visits at 6-month intervals for biospecimen collection and physical examinations, and to respond to questionnaires on sociodemographic, behavioral, and health information, with the same visit structure continued annually in the MWCCS. From April 2017–March 2018, participants in the MACS completed a pulmonary study visit to perform PFTs including pre-and post-bronchodilator spirometry and DLCO [26], and completion of the St. George’s Respiratory Questionnaire (SGRQ) [27]. From April 2018–January 2020, WIHS participants completed a pulmonary visit with similar data collection; DLCO was measured at a subset of six sites. A second pulmonary visit was completed among MWCCS participants between December 2021–January 2025. As described below, all available blood samples collected at MWCCS study visits between November 2020–August 2023 were tested for serological evidence of past SARS-CoV-2 infection.

The cohort for this analysis included MWCCS enrollees with serological evidence of a past SARS-CoV-2 infection who attended a pulmonary visit before SARS-CoV-2 infection and a pulmonary visit at least 6 months after first positive SARS-CoV-2 serology. To compare changes in pulmonary function by HIV serostatus within biological sex, we divided the overall cohort into four groups: (1) men with HIV (MWH) (2), men without HIV (MWoH) (3), women with HIV (WWH), and (4) women without HIV (WWoH).

### Ethical approval and informed consent

Written informed consent was obtained from MACS, WIHS, and MWCCS participants. The study was conducted in compliance with United States Health and Human Services human subjects protection requirements and Good Clinical Practice standards. The individual institutional review boards (IRBs) of all participating clinical centers approved all study protocols, and all participants provided written informed consent.

### Measures

#### Baseline characteristics

For all variables, we used the most recent recorded value prior to or on the date of the first positive SARS-CoV-2 serology. Vaccination status, prior hospitalization and diagnoses of asthma and COPD were determined via self-report (see online supplement). Criteria used to determine the presence of other comorbidities and clinical characteristics are included in the online supplement.

#### SARS-CoV-2 serology

All MWCCS visits occurring between November 2020 and August 2023 with available blood samples were tested for evidence of past SARS-CoV-2 infection using enzyme-linked immunosorbent assays that measured antibodies targeting the receptor-binding domain (RBD) of the spike protein and the full-length nucleocapsid protein, a component of the virus not included in the vaccine formulation available to the study participants [28–30]. Additional details related to assay conduct are included in the online supplement. Participants were categorized as having past SARS-CoV-2 infection if both anti-spike total IgG RBD and anti-nucleocapsid IgG thresholds for seropositivity were met. Participants demonstrating anti-spike total IgG RBD seronegativity but anti-nucleocapsid IgG seropositivity (1.2% of test results in the total MWCCS cohort) were excluded from analyses as this serological combination could represent either SARS-CoV-2 infection or false positive nucleocapsid antibody.

#### Pulmonary visit data collection

At each PFT visit, spirometry measures included FEV_1_ and FVC (EasyOne Pro or Easy on-PC, ndd Medical Technologies, Zurich, Switzerland), and were performed pre- and post-bronchodilator (BD) using inhalation of 360 ug of albuterol from a metered-dose inhaler [26, 31]. DLCO assessment followed spirometry measures. Quality for all PFTs was assessed by a central reading center per the American Thoracic Society/European Respiratory Society standards [32]. The online supplement describes PFT time point selection in further detail. We calculated PFT predicted values that accounted for age, sex, and height using race-neutral equations for spirometry [33] and DLCO [34]. DLCO values were adjusted for hemoglobin and carboxyhemoglobin obtained at the time of DLCO testing. Respiratory health status was assessed using the SGRQ [27]. The SGRQ is a 50-item questionnaire with three domains (symptoms, activity, impact) and a total score range of 0-100, with higher scores indicating worse status. The minimal clinically important difference (MCID) for the total score is four points [35].

### Statistical analysis

We calculated descriptive statistics for baseline characteristics by HIV serostatus: counts and proportions for categorical variables and means and standard deviations or median and interquartile range (IQR) for continuous variables, including baseline PFT measures. The primary outcomes for this analysis were the annualized change in each PFT measure (FEV_1_, FVC, and DLCO). FEV_1_ and FVC were reported using change in absolute ml/year and separately change in % predicted/year. For DLCO, only change in % predicted/year was reported. Annualized change was calculated as the difference between the post- and pre-SARS-CoV-2 infection measurement divided by the number of years between the tests. Density plots were used to visualize these changes for each type of PFT measure by HIV serostatus and mean change with 95% confidence intervals (CIs) were computed using the annualized change contributed by each person for each PFT measure.

We used linear regression to assess differences in annualized changes of post-BD FEV_1_, post-BD FVC, and DLCO by HIV serostatus, where the coefficient for HIV serostatus represented the mean difference in PFT measure changes between PWH and PWoH. Primary analyses were unadjusted for baseline covariates. Additional models adjusted for (1) baseline lung function, (2) current smoking status, and (3) baseline lung function and current smoking status. We also examined differences by HIV serostatus in the change of each pulmonary function measure within the following subgroups: participants who reported current, former, or never smoking; participants > 65 years old; participants diagnosed with asthma or COPD; participants with a body mass index (BMI) > 30 kg/m^2^; participants with a report of prior hospitalization; and participants with a report of prior SARS-CoV-2 vaccination (receipt of at least one dose of vaccine). All analyses were stratified by biological sex given different underlying characteristics of the MACS and WIHS participants.

We calculated change in SGRQ domains and total score by taking the mean difference between the score measured post-SARS-CoV-2 infection and the score measured pre-SARS-CoV-2 infection, reporting mean change with 95% CIs for each domain by HIV serostatus. We categorized individuals as having worsening of SGRQ domains if score increased by four or more points between first and second assessment.

All analyses were conducted using R (version 4.4.1).

## Results

### Study cohort

Of the 5220 enrolled MWCCS participants, a total of 778 participants (15%; 204 men and 574 women) had serological evidence of past SARS-CoV-2 infection along with acceptable PFT measurements before and after SARS-CoV-2 infection (Supplementary Figure S1). Supplementary Table S1 summarizes the availability of serologic and pre-SARS-CoV-2 exposure pulmonary function measures among participants. Of the 204 men included, 108 (53%) were MWH. Of the 574 women included, 404 (70%) were WWH. Baseline demographic and clinical characteristics are displayed in Table 1. Compared to MWoH, MWH were younger (median age 58 vs. 65 years) and a higher proportion self-identified as Black (41% vs. 22%), were current smokers (19% vs. 8%), reported low household income (27% vs. 10%), lower proportion obese (25% vs. 29%), and had prevalent comorbidities. Among women, there were no substantial differences by HIV serostatus in age, race, household income, obesity, or comorbidities. WWH were less likely to be current smokers than WWoH (22% vs. 35%). MWH and WWH had median CD4 cell count of 686 and 770 cells/mm^3^, respectively. In both men and women, approximately 75% of PWH had undetectable HIV viral load. Report of SARS-CoV-2 vaccine receipt was high among men (89% MWH, 95% MWoH) and lower among women (70% WWH, 71% WWoH). Report of hospitalization was 11% among MWH, 7% among MWoH, 7% among WWH, and 8% among WWoH.

**Table 1:** Cohort clinical characteristics

	Men		Women		
	MWH *N* = 108	MWoH *N* = 96	WWH *N* = 404		WWoH *N* = 170
Demographic Characteristics					
Age, median (Q1, Q3)	58 (52, 64)	65 (59, 69)	53 (47, 59)		52 (42, 58)
Race and ethnicity, n (%)					
Black, non-Hispanic	44 (40.7)	21 (21.9)	325 (80.4)		134 (78.8)
White, non-Hispanic	41 (38.0)	69 (71.9)	27 (6.7)		8 (4.7)
Another race, non-Hispanic	4 (3.7)	2 (2.1)	15 (3.7)		3 (1.8)
Any race, Hispanic	19 (17.6)	4 (4.2)	37 (9.2)		25 (14.7)
Annual household income ≤ $18,000, n (%)	28 (27.2)	9 (10.2)	197 (53.5)		83 (50.6)
Region, n (%)					
West	28 (25.9)	16 (16.7)	9 (2.2)		13 (7.6)
Northeast	0 (0.0)	1 (1.0)	136 (33.7)		64 (37.6)
Mid-Atlantic	39 (36.1)	26 (27.1)	29 (7.2)		10 (5.9)
South	0 (0.0)	0 (0.0)	190 (47.0)		66 (38.8)
Midwest	41 (38.0)	53 (55.2)	40 (9.9)		17 (10.0)
Clinical Characteristics^a^					
SARS-CoV-2 vaccination, n (%)	87 (88.8)	90 (94.7)	279 (69.9)		120 (71.0)
Missing	10	1	5		1
Prior hospitalization, n (%)	11 (10.8)	6 (6.5)	29 (7.3)		13 (7.8)
Missing	6	3	9		4
Hypertension, n (%)	63 (58.3)	51 (53.7)	264 (65.5)		102 (60.0)
Diabetes, n (%)	35 (32.4)	15 (15.6)	116 (28.9)		41 (24.1)
Myocardial infarction, n (%)	2 (2.0)	1 (1.1)	8 (2.0)		4 (2.4)
COPD (ever), n (%)	5 (5.0)	4 (4.3)	38 (9.6)		17 (10.3)
Asthma (ever), n (%)	11 (10.9)	7 (7.4)	94 (23.7)		41 (24.8)
Kidney disease/renal failure, n (%)	8 (7.9)	1 (1.1)	13 (3.3)		3 (1.8)
Race-free eGFR < 60, n (%)	17 (17.2)	8 (8.7)	71 (18.3)		10 (6.2)
HIV viral load, n (%)					
Undetectable: no signal	57 (52.8)		244 (60.4)		
Undetectable: under lower limit	20 (18.5)		57 (14.1)		
Detectable	31 (28.7)		103 (25.5)		
CD4 cells/mm^3^, median (Q1, Q3)	686 (495, 913)		770 (573, 997)		
BMI, median (Q1, Q3)	27 (24, 30)	27 (25, 31)	33 (27, 39)		32 (27, 37)
Underweight (< 18.5)	3 (2.8)	0 (0.0)	0 (0.0)		2 (1.2)
Healthy weight (18.5 to < 25)	37 (34.3)	26 (27.1)	56 (13.9)		19 (11.2)
Overweight (25 to < 30)	41 (38.0)	42 (43.8)	98 (24.3)		42 (24.7)
Obese (30+)	27 (25.0)	28 (29.2)	250 (61.9)		107 (62.9)
Hepatitis C, n (%)	5 (4.6)	2 (2.1)	8 (2.0)		4 (2.4)
Hepatitis B, n (%)	7 (6.5)	1 (1.0)	5 (1.2)		1 (0.6)
Behavioral Characteristics					
Current tobacco smoking, n (%)	18 (18.9)	7 (8.0)	85 (22.1)		56 (35.2)
Ever tobacco smoking, n (%)	70 (64.8)	59 (61.5)	227 (56.2)		111 (65.3)
Pack-years, median (Q1, Q3)	15 (4, 32)	9 (4, 22)	7 (3, 16)		8 (4, 17)

*COPD* Chronic obstructive pulmonary disease, *eGFR* Estimated glomerular filtration rate, *Q1* First quartile, *Q3* Third quartile, *MWH* Men with HIV, *MWoH* Men without HIV, *WWH* Women with HIV, *WWoH* Women without HIV

^a^See methods and supplementary information for comorbidity definitions

### Pulmonary function testing prior to SARS-CoV-2 infection

In men, pre- and post-BD FEV_1_ and FVC were largely normal, with mean values in MWH and MWoH ranging from 94 to 102% predicted (Table 2). When compared to MWoH, MWH had lower pre- and post-BD FEV_1_% predicted (pre-BD: 93.8% vs. 98.6% predicted; post-BD: 96.1% vs. 100.9% predicted). Similar magnitudes of difference were seen in pre- and post-BD FVC% predicted. MWH had lower adjusted DLCO% predicted than MWoH (93.7% vs. 96.0% predicted). Women had lower pre- and post- BD FEV_1_ and FVC than men, with mean values in WWH and WWoH ranging from 80–87% predicted. When compared to WWoH, WWH had lower pre- and post-BD FEV_1_% predicted (pre-BD: 80.2% vs. 82.5% predicted; post-BD: 82.5% vs. 84.5% predicted). Similar magnitudes of difference were seen in pre- and post-BD FVC% predicted. WWH had lower adjusted DLCO% predicted than WWoH (89.9% vs. 93.6% predicted).

**Table 2:** Pulmonary function before SARS-CoV-2 infection by HIV serostatus and source cohort

	Men		Women	
	MWH *N* = 108	MWoH *N* = 96	WWH *N* = 404	WWoH *N* = 170
Pre-BD				
FEV_1_, L	3.3 (0.7)	3.2 (0.6)	2.1 (0.5)	2.2 (0.5)
FEV_1_% predicted	93.8 (15.8)	98.6 (15.9)	80.2 (15.9)	82.5 (16.2)
FVC, L	4.3 (1.0)	4.2 (0.7)	2.7 (0.6)	2.9 (0.6)
FVC % predicted	97.9 (15.1)	101.9 (14.8)	83.9 (15.0)	86.9 (15.0)
FEV_1_/FVC	0.8 (0.1)	0.8 (0.1)	0.8 (0.1)	0.8 (0.1)
Post-BD				
FEV_1_, L	3.3 (0.8)	3.3 (0.7)	2.2 (0.5)	2.3 (0.6)
FEV_1_% predicted	96.1 (17.2)	100.9 (17.0)	82.5 (16.1)	84.5 (17.8)
FVC, L	4.2 (1.0)	4.3 (0.8)	2.7 (0.6)	2.8 (0.6)
FVC % predicted	96.3 (16.4)	101.1 (15.0)	83.3 (14.7)	86.0 (15.7)
FEV_1_/FVC	0.8 (0.1)	0.8 (0.1)	0.8 (0.1)	0.8 (0.1)
Adjusted^a^ DLCO % predicted	93.7 (19.1)	96.0 (14.7)	89.9 (17.1)	93.6 (21.2)

All values mean (SD) unless otherwise indicated. *BD* Bronchodilator, *DLCO* Diffusing capacity of the lung for carbon monoxide, *FVC* Forced vital capacity, *FEV*_1_ Forced expiratory volume in one second, *MWH* Men with HIV, *MWoH* Men without HIV, *PFT* Pulmonary function test, *WWH* Women with HIV, *WWoH* Women without HIV

^a^Adjusted for hemoglobin and carboxyhemoglobin

Missingness ranges from 4% (MWoH FEV_1_) to 68% (WWH Adjusted DLCO). Full details are in Supplementary Table S1

### Change in pulmonary function after SARS-CoV-2 infection

**Fig. 1:**
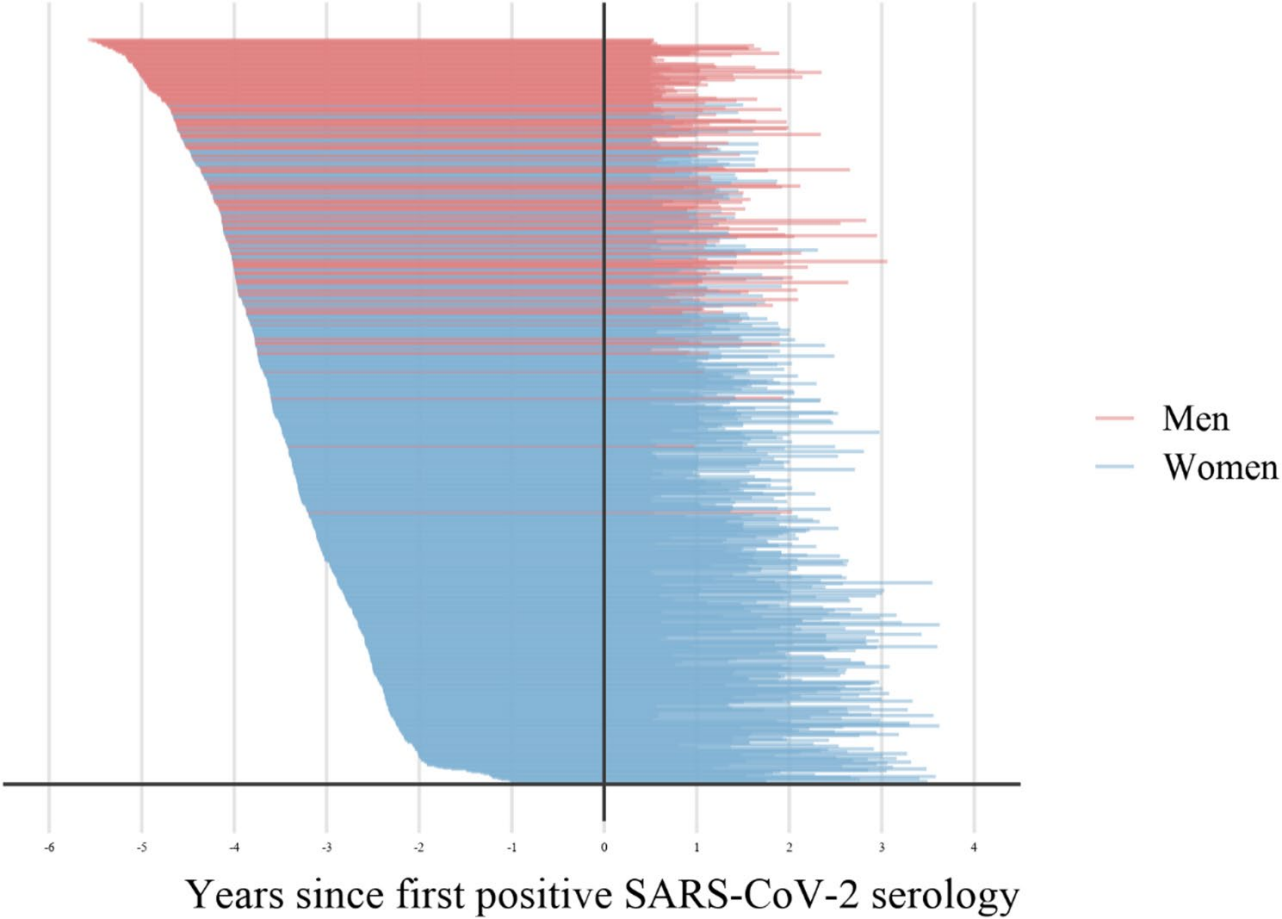
Timing of pulmonary function tests relative to first positive SARS-CoV-2 serology in men (red) and (blue) source cohorts

Figure 1 displays the timing of baseline and follow-up PFTs compared with the first positive SARS-CoV-2 antibody test. Among men, the median (IQR) time between PFTs was 66 (62–72) months for MWH and 68 (63–73) months for MWoH. The median (IQR) time from baseline PFT to first positive SARS-CoV-2 antibody test was 54 (49–59) months. The median (IQR) time from first positive SARS-CoV-2 antibody test to follow-up PFT was 13 9–17 months. Among women, the median (IQR) time between PFT tests was 60 (55–65) months for WWH and 58 (53–64) months for WWoH. The median (IQR) time from baseline PFT to first positive SARS-CoV-2 serology test was 39 (31–45) months. The median (IQR) time from the first positive SARS-CoV-2 serology test to follow-up PFT was 20 14–26 months.

**Fig. 2:**
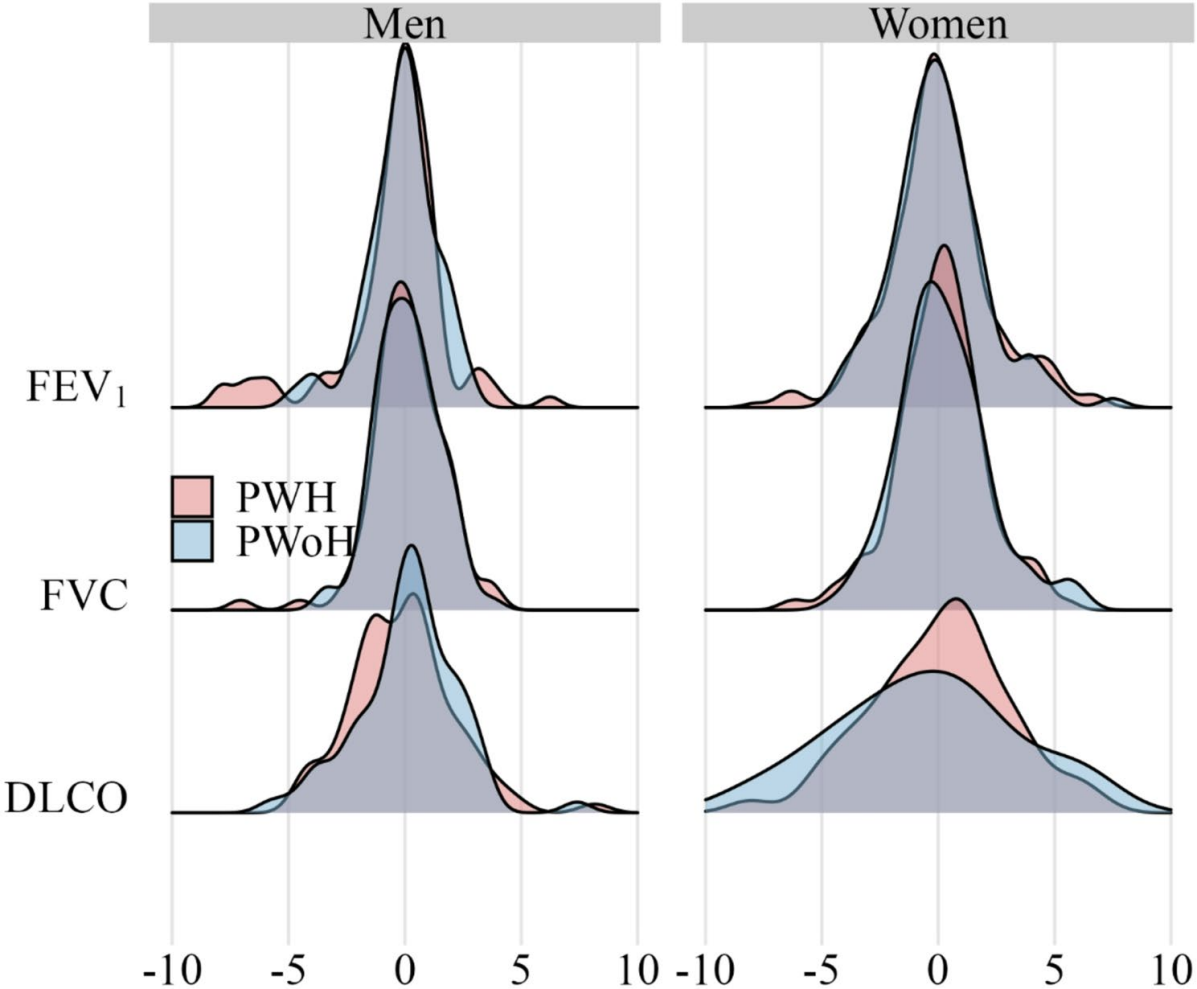
Distributions of annualized changes in percent predicted pulmonary function tests pulmonary function before and after SARS-CoV-2 infection by HIV serostatus (DLCO adjusted for hemoglobin and carboxyhemoglobin. *PWH* People with HIV, *PWoH* People without HIV, *DLCO* Diffusing capacity of the lungs for carbon monoxide)

The cohort-level distributions of post-BD FEV_1_, post-BD FVC, and DLCO change stratified by HIV serostatus are displayed in Fig. 2. The annualized change in absolute post-BD FEV_1_ was - 44.3 ml/year among MWH compared to -33.8 ml/year among MWoH [mean difference - 10.5 ml/year (95% CI -30.7 to 9.7) (Table 3). The annualized change in absolute post-BD FEV_1_ was - 19.8 ml/year among WWH compared to -14.8 ml/year among WWoH [mean difference - 5.0 ml/year (95% CI -18.2 to 8.2 )]. Changes in % predicted measures of FEV_1_ and FVC were similar. Among men, annualized change in % predicted DLCO was similar comparing MWH to MWoH [-0.1 vs. 0.1%/year; mean difference − 0.2%/year (95% CI -0.9 to 0.5)]. Similarly, among women annualized change in % predicted DLCO was not different comparing WWH to WWoH [0.1 vs. -0.1%/year; mean difference 0.1%/year (95% CI -1.2 to 1.4)]. Models adjusting for baseline FEV_1_ and current smoking status, separately or combined, yielded similar results (Supplementary Table S3, Supplementary Figures S8-S10).

**Table 3:** Annualized changes in pulmonary function, measured by FEV_1_, FVC, and DLCO, before and after SARS-CoV-2 infection, by HIV serostatus

	Men				Women			
	MWH Mean (95% CI)		MWoH Mean (95% CI)	Difference (95% CI)	WWH Mean (95% CI)		WWoH Mean (95% CI)	Difference (95% CI)
FEV_1_ ml/year								
Pre-BD	-35.6 (-48.1, -23.0)		-30.7 (-39.1, -22.3)	-4.8 (-20.2, 10.5)	-18.6 (-24.0, -13.1)		-17.4 (-25.6, -9.3)	-1.1(-11.0, 8.8)
Post-BD	-44.3 (-60.8, -27.8)		-33.8 (-44.5, -23.1)	-10.5 (-30.7, 9.7)	-19.8 (-27.0, -12.5)		-14.8 (-25.1, -4.5)	-5.0 (-18.2, 8.2)
FEV_1_% predicted/year								
Pre-BD	-0.3 (-0.7, 0.1)		-0.1 (-0.3, 0.2)	-0.2 (-0.7, 0.3)	0.0 (-0.2, 0.2)		0.0 (-0.3, 0.3)	-0.0 (-0.4, 0.4)
Post-BD	-0.5 (-0.9, 0.0)		-0.1 (-0.5, 0.2)	-0.3 (-0.9, 0.3)	-0.0 (-0.3, 0.2)		0.1 (-0.3, 0.5)	-0.2 (-0.7, 0.3)
FVC mL/year								
Pre-BD	-26.5 (-40.4, -12.7)		-17.2 (-28.1, -6.3)	-9.3 (-27.0, 8.3)	-17.3 (-23.8, -10.9)		-16.2 (-26.2, -6.3)	-1.1 (-12.9, 10.8)
Post-BD	-20.2 (-33.7, -6.7)		-22.7 (-34.3, -11.0)	2.5 (-15.5, 20.5)	-14.5 (-21.2, -7.8)		-10.2 (-21.7, 1.2)	-4.3 (-17.3, 8.8)
FVC % predicted/year								
Pre-BD	-0.0 (-0.4, 0.3)		0.2 (-0.0, 0.5)	-0.3 (-0.7, 0.2)	0.0 (-0.2, 0.3)		0.1 (-0.3, 0.4)	-0.0 (-0.4, 0.4)
Post-BD	0.1 (-0.2, 0.5)		0.1 (-0.2, 0.4)	0.0 (-0.4, 0.5)	0.1 (-0.1, 0.3)		0.2 (-0.1, 0.6)	-0.1 (-0.5, 0.3)
DLCO^a^ % predicted/year	-0.1 (-0.6, 0.4)		0.1 (-0.4, 0.6)	-0.2 (-0.9, 0.5)	0.1 (-0.5, 0.6)		-0.1 (-1.5, 1.4)	0.1 (-1.2, 1.4)

*BD* Bronchodilator, *CI* Confidence interval, *DLCO* Diffusing capacity of the lung for carbon monoxide, *FEV*_1_ Forced expiratory volume in one second, *FVC* Forced vital capacity, *MWH* Men with HIV, *MWoH* Men without HIV, *PFT* Pulmonary function test, *WWH* Women with HIV, *WWoH* Women without HIV

^a^Adjusted for hemoglobin and carboxyhemoglobin

### Pulmonary function sensitivity analyses

Stratifying cohorts by key demographic and clinical characteristics (age, smoking status, pre-existing obstructive lung disease, BMI, prior hospitalization, report of SARS-CoV-2 vaccine receipt) did not identify any factors consistently associated with differential annual change in FEV_1_ or FVC by HIV serostatus (Supplementary Figs. 2–5). There were no demographic or clinical characteristics associated with differential annual change in % predicted DLCO by HIV serostatus in men (Supplementary Fig. 6). Among women aged 65 + years, greater decline was seen among WWoH compared to WWH (mean difference 5.4%/year; 95% CI 3.0 to 7.7) (Supplementary Fig. 7). Within each cohort we compared the 10% with the largest decline in % predicted FEV_1_ to the remaining 90% of the cohort. Those with the largest decline in % predicted FEV_1_ were more likely to be current smokers, have lower income, and report prior hospitalization compared to the remaining 90% of individuals within each cohort. Additionally, among women, worst decliners were more likely to have obesity and chronic obstructive pulmonary disease (Supplementary Table S4).

### Respiratory symptoms prior to SARS-CoV-2 infection

Before SARS-CoV-2 infection, men had relatively mild respiratory health impairment, with mean values of SGRQ domains ranging from 5.2 to 17.9 among MWH and 2.7 to 12.8 among MWoH (Supplementary Table S2). All domains were higher (worse) among MWH compared to MWoH, with highest scores seen in the symptom domain (MWH: 17.9; MWoH 12.8). Among women, overall respiratory impairment was worse compared to men, with mean values of SGRQ domains ranging from 8.1 to 30.2 among WWH and 9.0 to 28.6 among WWoH. Among women, pre-positive-SARS-CoV-2 serology SGRQ domain scores were similar between HIV serostatus groups.

### Change in respiratory symptoms after SARS-CoV-2 infection

Among MWH and MWoH, neither individual SGRQ domains nor total score demonstrated a clinically significant change from the assessment prior to SARS-CoV-2-infection to the assessment after (Table 4). Moreover, there were no meaningful differences when comparing across serostatus groups. Similarly, among WWH and WWoH, none of the individual SGRQ domains or total score had a clinically significant change from the assessment prior to SARS-CoV-2 infection to the assessment after. Among women, the SGRQ activity domain had the most substantial change by HIV serostatus: in WWH, the activity score decreased (improved) by 3.1 points, while among WWoH the activity score increased (worsened) by 2.1 points [difference: -5.2 points (95% CI -10.0 to -0.4)]. Other domains and total score of the SGRQ among women did not differ comparing WWH to WWoH.

**Table 4:** Changes in St. George’s Respiratory Questionnaire domains comparing post- to pre- SARS-CoV-2 infection, with differences by HIV serostatus

Domain	Men			Women			
	Mean (SD)		Difference (95% CI)	Mean (SD)			Difference (95% CI)
	MWH	MWoH		WWH		WWoH	
Symptoms	-2.7 (21.7)	-0.1 (16.5)	-2.6 (-8.1, 2.9)	-1.9 (20.9)		-1.2 (23.6)	-0.7 (-4.6, 3.3)
Activity	2.0 (19.9)	0.1 (18.7)	1.9 (-3.6, 7.4)	-3.1 (27.0)		2.1 (25.2)	-5.2 (-10.0, -0.4)
Impacts	-0.4 (9.3)	-0.6 (8.2)	0.2 (-2.3, 2.7)	-0.3 (14.5)		-0.4 (13.0)	0.2 (-2.4, 2.7)
Total	0.1 (11.9)	-0.2 (10.7)	0.3 (-3.0, 3.5)	-1.8 (16.0)		-0.2 (15.2)	-1.6 (-4.5, 1.2)

*CI* Confidence interval, *MWH* Men with HIV, *MWoH* Men without HIV, *SD* Standard deviation, *WWH* Women with HIV, *WWoH* Women without HIV

**Fig. 3:**
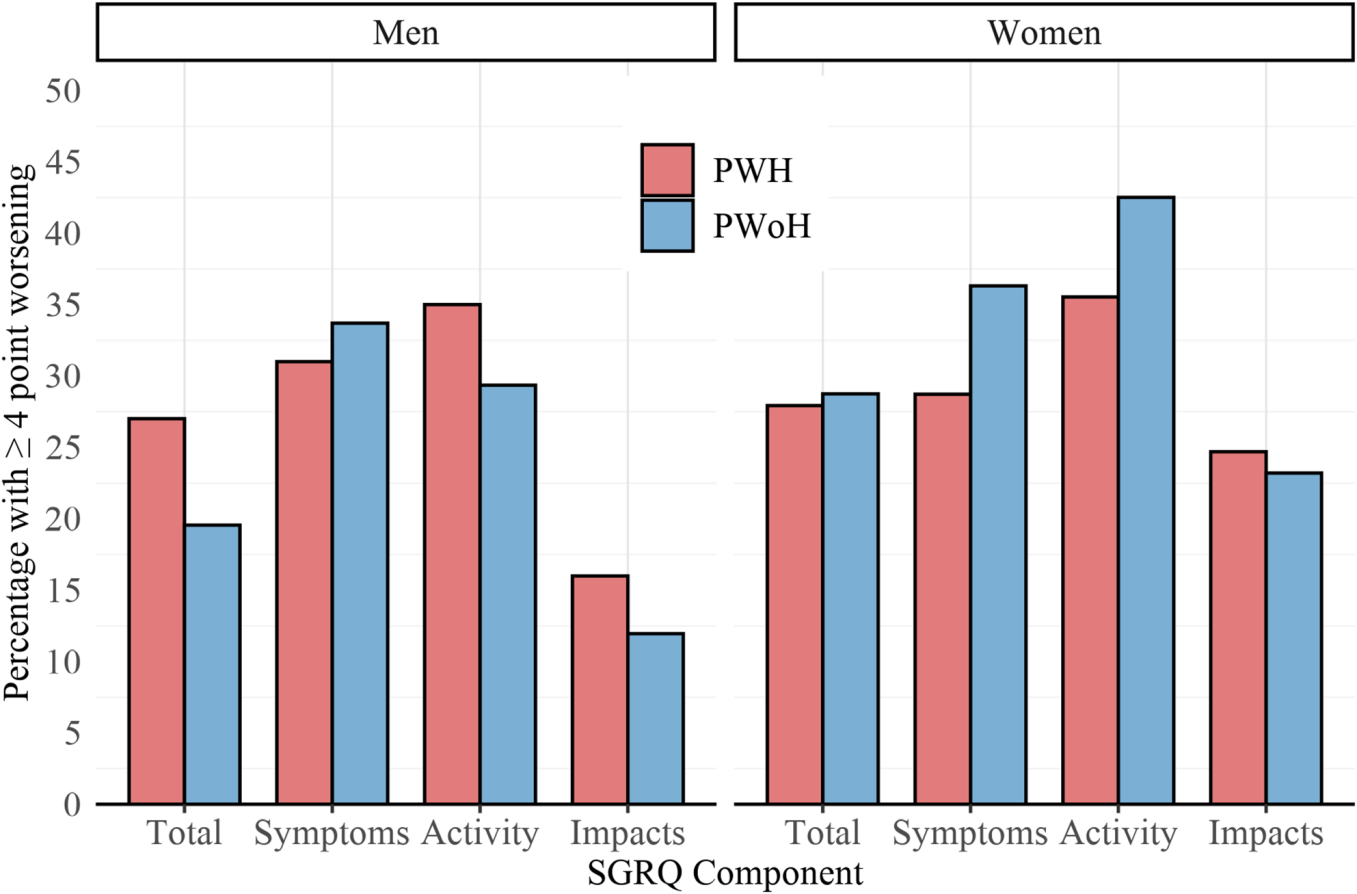
Percentage of people with SGRQ scores that worsen (increase) at least 4 points from pre-SARS-CoV-2 infection pulmonary visit to post-SARS-CoV-2 infection pulmonary visit, by sex and HIV serostatus. (*PWH* People with HIV, *PWoH* People without HIV, *SGRQ* St. George’s Respiratory Questionnaire)

The proportions of men and women experiencing a 4 or more-point increase (worsening) in SGRQ score domains or total score after infection with SARS-CoV-2 are displayed in Fig. 3 and summarized in Supplementary Table S5. There were numerically higher proportions of MWH compared to MWoH experiencing worsening total SGRQ (27% vs. 20%), activity (35% vs. 29%), and impact (16% vs. 12%) scores. Among women, the directionality was opposite, with numerically higher proportions of WWoH compared to WWH experiencing worsening symptoms (36% vs. 29%) and activity (43% vs. 35%) scores.

## Discussion

In this prospective analysis from the multisite MWCCS participants with pre- and post-SARS-CoV-2 infection pulmonary function testing and St. George’s Respiratory Questionnaire, we observed that among individuals with serological evidence of SARS-CoV-2 infection, there were no differences in pulmonary function changes comparing PWH to PWoH. Moreover, there were no consistent differences in changes in respiratory symptom burden comparing PWH to PWoH. These findings suggest that HIV infection is not an independent risk factor for worsening pulmonary outcomes following a SARS-CoV-2 infection.

In persons without HIV, findings on the impacts of SARS-CoV-2 infection on pulmonary function measurements are inconsistent. Lewis and colleagues observed that among a cohort of 80 individuals with mild to moderate COVID-19 disease, FEV_1_, FVC or DLCO measured within one year after SARS-CoV-2 infection did not differ from pre-infection values [36]. A separate analysis of 52 individuals from the Copenhagen General Population Study observed that individuals with mild COVID-19 infection had adjusted excess decline in FEV_1_ of 13.0 mL/year in the two years post-infection compared to uninfected participants [37]. The RECoVERED Study group reported that among a cohort of 301 individuals, one year after hospitalized or non-hospitalized SARS-CoV-2 infection, 25% of participants had impaired pulmonary function [38]. To our knowledge, our analysis represents the first and the largest description of changes in objective and subjective pulmonary measures comparing PWH and PWoH with serological evidence of SARS-CoV-2 infection. We add to the literature our findings that there was no observe differences in longitudinal pulmonary outcomes comparing PWH to PWoH with median follow-up times of 13 and 20 months after SARS-CoV-2 infection in men and women, respectively.

In the MWCCS, the annual change in absolute FEV_1_ among PWoH with serological evidence of SARS-CoV-2 infection ranged from - 15 to -34 ml/year, and when measured using % predicted values ranged 0.1% to -0.1% predicted. These ranges are similar to the unadjusted magnitude of change reported by Lewis et al. (0.2% predicted) but are less than those reported in the two-year follow-up study by Iversen et al. (-46 ml/year). The lack of longitudinal change in DLCO % predicted observed in our analysis also aligns with the reports of Lewis and Iversen. Taken together, the pulmonary function trajectories observed in our SARS-CoV-2 seropositive PWoH group align with those reported in other cohorts of PWoH.

The lack of differential pulmonary function changes among PWH has several potential explanations. First, HIV infection simply may not be an independent risk factor for worse pulmonary outcomes after SARS-CoV-2 infection. It is noteworthy that the majority of MWCCS participants contributing data to this analysis have well-controlled HIV as measured by an undetectable HIV viral load and robust CD4 cell count. The additive impact of HIV infection on the background risk of accelerated pulmonary decline following SARS-CoV-2 infection may be attenuated with effective HIV treatment. The distribution of FEV_1_ decline in MWH has more outliers with reduced lung function. It is possible that among individuals with uncontrolled HIV, a subset experiences greater declines in pulmonary function measures compared to PWoH, but we are underpowered to study this hypothesis in our cohort. A second possible explanation for our findings is that the duration of follow-up between SARS-CoV-2 infection and post-infection PFT may have been insufficient to detect longitudinal changes. However, in the study by Iversen et al., differences in pulmonary function between those with and without SARS-CoV-2 infection were observed as early as six months, with changes persisting up to 24 months, making the duration of follow-up less likely a factor. Third, the wide range of time between the first positive SARS-CoV-2 serology and the follow-up PFT in this observational cohort study may have impacted the ability to detect differences in pulmonary function changes between PWH and PWoH. Studies have reported an initial decline in pulmonary function early post-infection that recovers one year after infection [39, 40], highlighting the dynamic nature of lung damage and recovery after SARS-CoV-2 infection. Finally, the MWCCS participants included in this analysis had largely normal pulmonary function prior to SARS-CoV-2 infection. Pre-existing lung disease is associated with worse long-term pulmonary outcomes after SARS-CoV-2 infection [41]. It is possible that a similar analysis in a cohort of individuals with and without HIV, and with pre-existing lung disease, would yield different results.

Our findings of no differences in change in respiratory symptom burden after SARS-CoV-2 infection between PWH to PWoH aligns with our pulmonary function findings. The overall symptom burden in the analytical cohort prior to SARS-CoV-2 infection was low, affording the opportunity to detect worsening symptoms after SARS-CoV-2 infection. Despite this, we did not see any consistent trends in the worsening or improving of respiratory symptoms after SARS-CoV-2 infection. Similar to our pulmonary function findings, the lack of associations between HIV serostatus and changes in respiratory symptoms after SARS-CoV-2 infection may be due to a lack of biological impact of HIV infection on symptom domains or related to the specific characteristics of the MWCCS analytical cohort.

This analysis has limitations. The timing of PFT assessment before and after SARS-CoV-2 serological testing was variable in the setting of an observational cohort study. The variable time windows create heterogeneity in our assessments of the impacts of SARS-CoV-2 infection on longitudinal outcomes. While we did not observe an impact of hospitalization on pulmonary outcomes, the MWCCS did not systematically collect data on the presence of pneumonia nor the severity or treatment of SARS-CoV-2 infection among all participants, an important potential modifier of longitudinal pulmonary outcomes. Therefore, it is possible our findings would differ if we were able to better assess severity of SARS-CoV-2 infection (e.g., people requiring oxygen support). Moreover, this analysis is only among those individuals who returned for a MWCCS visit; hence, excluding individuals who may have died due to SARS-CoV-2 infection. Due to factors such as antibody fading and asymptomatic infection, we were not able to accurately identify individuals with no history of SARS-CoV-2 infection to compare lung function changes between those with and without SARS-CoV-2 infection. Finally, the analytical cohort was comprised largely of PWH who had controlled HIV infection. This prevents the generalization of our findings to populations where access to HIV treatment is poor.

Despite these limitations, our study has several strengths. First, the MWCCS is a multicenter cohort with well-characterized participants including epidemiologically well-matched comparator PWoH. This cohort affords the important opportunity to robustly isolate the potential impacts of HIV infection on pulmonary outcomes. Second, we determined SARS-CoV-2 infection using a biological marker (positive serology) rather than self-report. This approach permitted the inclusion of asymptomatic infections, which are largely excluded from prior reports of hospitalized and treated individuals. Finally, the collection of both physiologic and patient-reported pulmonary domains permitted a comprehensive analysis of the impacts of HIV infection on pulmonary outcomes after SARS-CoV-2 infection.

## Conclusions

In conclusion, our study found that among individuals with serological evidence of SARS-CoV-2 infection, PWH did not have differential longitudinal changes in objective or subjective changes in pulmonary outcomes compared to PWoH. This study suggests that the long-term respiratory impacts of SARS-CoV-2 infection may not be worse in PWH than in PWoH. Further research is needed to understand how uncontrolled HIV infection and severity of SARS-CoV-2 infection may increase risks of adverse pulmonary outcomes following a SARS-CoV-2 infection.

## Supplementary Information

Supplementary Material 1.

## Data Availability

Access to individual-level data from the MACS/WIHS Combined Cohort Study Data (MWCCS) may be obtained upon review and approval of a MWCCS concept sheet. Links and instructions for online concept sheet submission are on the study website (https:/statepi.jhsph.edu/mwccs/work-with-us) .

## Abbreviations

ART: Antiretroviral therapy
BD: Bronchodilator
BMI: Body mass index
CI: Confidence interval
COPD: Chronic obstructive pulmonary disease
COVID-19: Coronavirus disease 2019
DLCO: Diffusing capacity for carbon monoxide
FEV_1_: Forced expiratory volume in one second
FVC: Forced vital capacity
IQR: Interquartile range
MACS: Multicenter AIDS Cohort Study
MCID: Minimal clinically important difference
MWCCS: MACS/WIHS Combined Cohort Study
MWH: Men with HIV
MWoH: Men without HIV
PFT: Pulmonary function testing
PWH: People with HIV
PWoH: People without HIV
SARS-CoV-2: Severe acute respiratory syndrome coronavirus-2
SGRQ: St. George’s Respiratory Questionnaire
WIHS: Women’s Interagency HIV Study
WWH: Women with HIV
WWoH: Women without HIV

## References

[1] Li Q, Guan X, Wu P, Wang X, Zhou L, Tong Y, et al. Early transmission dynamics in Wuhan, China, of novel Coronavirus-Infected pneumonia. N Engl J Med. 2020;382(13):1199–207. 31995857 10.1056/NEJMoa2001316 PMC7121484

[2] Kamel Boulos MN, Geraghty EM. Geographical tracking and mapping of coronavirus disease COVID-19/severe acute respiratory syndrome coronavirus 2 (SARS-CoV-2) epidemic and associated events around the world: how 21st century GIS technologies are supporting the global fight against outbreaks and epidemics. Int J Health Geogr. 2020;19(1):8. 32160889 10.1186/s12942-020-00202-8 PMC7065369

[3] Johnson KD, Harris C, Cain JK, Hummer C, Goyal H, Perisetti A. Pulmonary and Extra-Pulmonary clinical manifestations of COVID-19. Front Med (Lausanne). 2020;7:526. 32903492 10.3389/fmed.2020.00526 PMC7438449

[4] Singh SJ, Baldwin MM, Daynes E, Evans RA, Greening NJ, Jenkins RG, et al. Respiratory sequelae of COVID-19: pulmonary and extrapulmonary origins, and approaches to clinical care and rehabilitation. Lancet Respir Med. 2023;11(8):709–25. 37216955 10.1016/S2213-2600(23)00159-5 PMC10198676

[5] Toh MR, Teo YR, Poh LCR, Tang Y, Soh RY, Sharma K, et al. Impact of COVID infection on lung function test and quality of life. Sci Rep. 2023;13(1):17275. 37828107 10.1038/s41598-023-43710-w PMC10570308

[6] Huntley CC, Patel K, Bil Bushra SE, Mobeen F, Armitage MN, Pye A et al. Pulmonary function test and computed tomography features during follow-up after SARS, MERS and COVID-19: a systematic review and meta-analysis. ERJ Open Res. 2022;8(2):00056-2022. 10.1183/23120541.00056-2022 PMC9035766 35642193

[7] Lee JH, Yim JJ, Park J. Pulmonary function and chest computed tomography abnormalities 6–12 months after recovery from COVID-19: a systematic review and meta-analysis. Respir Res. 2022;23(1):233. 36068582 10.1186/s12931-022-02163-x PMC9446643

[8] Wit F, Reiss P, Rijnders B, Rokx C, Roukens A, Brinkman K, et al. COVID-19 in people with HIV in the Netherlands. AIDS. 2023;37(11):1671–81. 37199566 10.1097/QAD.0000000000003597 PMC10399951

[9] Thornhill J, Orkin C, Cevik M. Estimating the global impact of coronavirus disease 2019 on people living with HIV. Curr Opin Infect Dis. 2023;36(1):20–5. 36729763 10.1097/QCO.0000000000000898

[10] Rodriguez-Miguelez P, Heefner A, Carbone S. Recognizing risk factors associated with poor outcomes among patients with COVID-19. Prog Cardiovasc Dis. 2023;76:3–11. 36693489 10.1016/j.pcad.2023.01.006 PMC9862711

[11] Venturas JP. HIV and COVID-19 disease. Semin Respir Crit Care Med. 2023;44(1):35–49. 36646084 10.1055/s-0042-1758852

[12] Miller KW, Gandhi RT. The severity of COVID-19 across the spectrum of HIV. Curr Opin HIV AIDS. 2023;18(3):119–25. 37144613 10.1097/COH.0000000000000791 PMC10321774

[13] Popescu I, Drummond MB, Gama L, Coon T, Merlo CA, Wise RA, et al. Activation-induced cell death drives profound lung CD4(+) T-cell depletion in HIV-associated chronic obstructive pulmonary disease. Am J Respir Crit Care Med. 2014;190(7):744–55. 25137293 10.1164/rccm.201407-1226OC PMC4299615

[14] Konstantinidis I, Crothers K, Kunisaki KM, Drummond MB, Benfield T, Zar HJ, et al. HIV-associated lung disease. Nat Rev Dis Primers. 2023;9(1):39. 37500684 10.1038/s41572-023-00450-5 PMC11146142

[15] Wang RJ, Nouraie M, Kunisaki KM, Huang L, Tien PC, Anastos K, et al. Lung function in women with and without human immunodeficiency virus. Clin Infect Dis. 2023;76(3):e727–35. 35604821 10.1093/cid/ciac391 PMC9907549

[16] Konstantinidis I, Qin S, Fitzpatrick M, Kessinger C, Gentry H, McMahon D, et al. Pulmonary function trajectories in people with HIV: analysis of the Pittsburgh HIV lung cohort. Ann Am Thorac Soc. 2022;19(12):2013–20. 35939796 10.1513/AnnalsATS.202204-332OC PMC9743474

[17] Verboeket SO, Boyd A, Wit FW, Verheij E, van der Schim MF, Kootstra N, et al. Changes in lung function among treated HIV-positive and HIV-negative individuals: analysis of the prospective AGE(h)IV cohort study. Lancet Healthy Longev. 2021;2(4):e202–11. 36098121 10.1016/S2666-7568(21)00033-7

[18] Ronit A, Benfield T, Lundgren J, Vestbo J, Afzal S, Nordestgaard BG, et al. Interstitial lung abnormalities in people with HIV infection and uninfected controls. J Infect Dis. 2020;221(12):1973–7. 32002544 10.1093/infdis/jiaa047

[19] Konstantinidis I, Zou RH, Papageorgiou SN, Ronit A, Drummond MB, Kunisaki KM, et al. Effect of human immunodeficiency virus on lung function and structure: A systematic review and Meta-Analysis. Ann Am Thorac Soc. 2025;22(2):274–84. 39417747 10.1513/AnnalsATS.202404-384OC PMC11808551

[20] Meiring S, Tempia S, Bhiman JN, Buys A, Kleynhans J, Makhasi M, et al. Prolonged shedding of severe acute respiratory syndrome coronavirus 2 (SARS-CoV-2) at high viral loads among hospitalized immunocompromised persons living with human immunodeficiency virus (HIV), South Africa. Clin Infect Dis. 2022;75(1):e144–56. 35134129 10.1093/cid/ciac077 PMC8903337

[21] Augello M, Bono V, Rovito R, Tincati C, Marchetti G. Immunologic interplay between HIV/AIDS and COVID-19: adding fuel to the flames? Curr HIV/AIDS Rep. 2023;20(2):51–75. 36680700 10.1007/s11904-023-00647-z PMC9860243

[22] Kumar R, Aktay-Cetin O, Craddock V, Morales-Cano D, Kosanovic D, Cogolludo A, et al. Potential long-term effects of SARS-CoV-2 infection on the pulmonary vasculature: multilayered cross-talks in the setting of coinfections and comorbidities. PLoS Pathog. 2023;19(1):e1011063. 36634048 10.1371/journal.ppat.1011063 PMC9836319

[23] Kaslow RA, Ostrow DG, Detels R, Phair JP, Polk BF, Rinaldo CR. Jr. The multicenter AIDS cohort study: rationale, organization, and selected characteristics of the participants. Am J Epidemiol. 1987;126(2):310–8. 3300281 10.1093/aje/126.2.310

[24] Adimora AA, Ramirez C, Benning L, Greenblatt RM, Kempf MC, Tien PC, et al. Cohort profile: the women’s interagency HIV study (WIHS). Int J Epidemiol. 2018;47(2):393–i4. 29688497 10.1093/ije/dyy021 PMC5913596

[25] D’Souza G, Bhondoekhan F, Benning L, Margolick JB, Adedimeji AA, Adimora AA, et al. Characteristics of the MACS/WIHS combined cohort study: opportunities for research on aging with HIV in the longest US observational study of HIV. Am J Epidemiol. 2021;190(8):1457–75. 33675224 10.1093/aje/kwab050 PMC8484936

[26] Drummond MB, Edmonds A, Ramirez C, Stosor V, Barjaktarevic IZ, Morris A, et al. Association between HIV and prevalence and manifestations of asthma: analysis of the multicenter AIDS cohort study and women’s interagency HIV study. J Acquir Immune Defic Syndr. 2022;91(5):419–28. 36083508 10.1097/QAI.0000000000003088 PMC9649933

[27] Jones PW, Quirk FH, Baveystock CM, Littlejohns P. A self-complete measure of health status for chronic airflow limitation. The St. George’s respiratory questionnaire. Am Rev Respir Dis. 1992;145(6):1321–7. 1595997 10.1164/ajrccm/145.6.1321

[28] Fox T, Geppert J, Dinnes J, Scandrett K, Bigio J, Sulis G, et al. Antibody tests for identification of current and past infection with SARS-CoV-2. Cochrane Database Syst Rev. 2022;11(11):CD013652. 36394900 10.1002/14651858.CD013652.pub2 PMC9671206

[29] Premkumar L, Segovia-Chumbez B, Jadi R, Martinez DR, Raut R, Markmann A et al. The receptor binding domain of the viral Spike protein is an immunodominant and highly specific target of antibodies in SARS-CoV-2 patients. Sci Immunol. 2020;5(48):eabc8413. 10.1126/sciimmunol.abc8413 PMC7292505 32527802

[30] Narowski TM, Raphel K, Adams LE, Huang J, Vielot NA, Jadi R, et al. SARS-CoV-2 mRNA vaccine induces robust specific and cross-reactive IgG and unequal neutralizing antibodies in Naive and previously infected people. Cell Rep. 2022;38(5):110336. 35090596 10.1016/j.celrep.2022.110336 PMC8769879

[31] Kunisaki KM, Nouraie M, Jensen RL, Chang D, D’Souza G, Fitzpatrick ME, et al. Lung function in men with and without HIV. AIDS. 2020;34(8):1227–35. 32287070 10.1097/QAD.0000000000002526 PMC7362901

[32] Miller MR, Hankinson J, Brusasco V, Burgos F, Casaburi R, Coates A, et al. Standardisation of spirometry. Eur Respir J. 2005;26(2):319–38. 16055882 10.1183/09031936.05.00034805

[33] Bowerman C, Bhakta NR, Brazzale D, Cooper BR, Cooper J, Gochicoa-Rangel L, et al. A Race-neutral approach to the interpretation of lung function measurements. Am J Respir Crit Care Med. 2023;207(6):768–74. 36383197 10.1164/rccm.202205-0963OC

[34] Stanojevic S, Graham BL, Cooper BG, Thompson BR, Carter KW, Francis RW et al. Official ERS technical standards: global lung function initiative reference values for the carbon monoxide transfer factor for Caucasians. Eur Respir J. 2017;50(3):1700010. 10.1183/13993003.00010-2017 28893868

[35] Jones PW. Interpreting thresholds for a clinically significant change in health status in asthma and COPD. Eur Respir J. 2002;19(3):398–404. 11936514 10.1183/09031936.02.00063702

[36] Lewis KL, Helgeson SA, Tatari MM, Mallea JM, Baig HZ, Patel NM. COVID-19 and the effects on pulmonary function following infection: A retrospective analysis. EClinicalMedicine. 2021;39:101079. 34405138 10.1016/j.eclinm.2021.101079 PMC8360709

[37] Iversen KK, Ronit A, Ahlstrom MG, Nordestgaard BG, Afzal S, Benfield T. Lung function trajectories in mild COVID-19 with 2-year Follow-up. J Infect Dis. 2024;229(6):1750–8. 38271235 10.1093/infdis/jiae037

[38] van Willigen HDG, Wynberg E, Verveen A, Dijkstra M, Verkaik BJ, Figaroa OJA, et al. One-fourth of COVID-19 patients have an impaired pulmonary function after 12 months of disease onset. PLoS ONE. 2023;18(9):e0290893. 37695755 10.1371/journal.pone.0290893 PMC10495003

[39] Suppini N, Fira-Mladinescu O, Traila D, Motofelea AC, Marc MS, Manolescu D et al. Longitudinal analysis of pulmonary function impairment one year Post-COVID-19: A Single-Center study. J Pers Med. 2023;13(8):1190. 10.3390/jpm13081190 PMC10455572 37623441

[40] Zhang H, Li X, Huang L, Gu X, Wang Y, Liu M, et al. Lung-function trajectories in COVID-19 survivors after discharge: A two-year longitudinal cohort study. EClinicalMedicine. 2022;54:101668. 36188433 10.1016/j.eclinm.2022.101668 PMC9514976

[41] Mara G, Nini G, Cotoraci C. Impact of pulmonary comorbidities on COVID-19: acute and Long-Term evaluations. J Clin Med. 2025;14(5):1446. 10.3390/jcm14051446 PMC11900502 40094893

