# Supplementary material for: Associations between serological evidence of SARS-CoV-2 infection and longitudinal pulmonary outcomes among people with HIV: analysis of the MACS/WIHS combined cohort study (MWCCS)
Source: Respir Res. 2026 Feb 5;27:119. doi: 10.1186/s12931-026-03542-4 (PMC12964756; doi:10.1186/s12931-026-03542-4)
Supplement: Supplementary file 1 — Supplementary Material 1. [file 12931_2026_3542_MOESM1_ESM.docx]

Associations between serological evidence of SARS-CoV-2 infection and longitudinal pulmonary outcomes among people with HIV:

Analysis of the MACS/WIHS Combined Cohort Study (MWCCS)

Supplementary File

Methods

Criteria to determine presence of comorbidities

| Comorbidity | Definition |
| --- | --- |
| Asthma/COPD | Participant self-report at MWCCS visits prior to the first SARS-CoV-2 positive serology of having ever received an asthma or COPD diagnosis. |
| Hypertension | SBP ≥ 130 or DBP ≥ 80, or use of hypertensive medications with ever self-reported diagnosis |
| Diabetes | Fasting glucose ≥ 126 mg/dL confirmed if diabetes medication reported at a subsequent visit or second fasting glucose ≥ 126 or HbA1C ≥ 6.5% for the participant OR Ever self-reported DM confirmed if diabetes medications reported or two fasting glucose ≥ 126 or (concurrent HbA1C ≥ 6.5% and fasting glucose ≥ 126) for the participant OR HbA1C ≥ 6.5% confirmed if diabetes medications reported or fasting glucose ≥ 126 for the participant. DIABETES will become as yes thereafter; this DM definition is only defined for women who are not pregnant at the visit |
| Hepatitis C | Acute infection (HCV RNA+, < 1 year) or chronic infection (HCV RNA+, ≥ year) |
| Hepatitis B | Determined by HBsAg and HBcaB: acute (HBsAg+, < 1 year) or chronic infection (HBsAg+ ≥ 1 year) |
| Smoking pack-years | cumulative pack years, if not reported at most recent visit is last observation carried forward. Ever smokers include current smokers, former smokers, and “discrepant” smokers |

SARS-CoV-2 Serological Testing

Enzyme-Linked Immunosorbent Assays that measured antibodies targeting the receptor-binding domain (RBD) of the spike protein and the full length nucleocapsid protein were used for determination of SARS-CoV-2 serological testing.(1, 2) To measure spike RBD total Ig, 50 μL of streptavidin (4 μg/mL in Tris-Buffered Saline (TBS), pH 7.4) was coated in a high-binding microtiter plate and followed by blocking solution (1:1 Non-Animal Protein-BLOCKER™ in TBS). Heat-inactivated serum samples were diluted at 1:40 in TBS-based diluent buffer (TBS + 0.05% Tween-20) containing 3% bovine serum albumin (BSA) and 1µg/ml biotinylated spike RBD was added. The plate was washed, and then 50 μL of horseradish peroxidase-conjugated Goat Anti-Human secondary antibody (IgG, IgM and IgA) was added. After washing, 50 μL of 3,3′,5,5′-Tetramethylbenzidine substrate (Sigma-Aldrich) was added, and absorbance was measured at 450 nm following the addition of 50 μL of 1 N HCl stop solution. To measure Nucleocapsid Ab, 50 μL anti-maltose-binding protein (MBP) (New England Biolabs) at 3 μg/mL in TBS pH 7.4 was coated and then blocked with 100 μL of blocking solution (3% non-fat milk in TBST). 50 μL of 2 μg/mL MBP fused full-length SARS-CoV-2 nucleocapsid or MBP protein in blocking solution were added to adjacent wells. Heat-inactivated serum at 1:40 dilution was added to the plate. After incubating the plate with alkaline phosphatase-conjugated secondary goat anti-human IgG, p-Nitrophenyl phosphate substrate (SIGMA FAST) was added to the plate, and absorbance was measured at 405 nm. Background signals arising from the MBP-coated wells were subtracted from the nucleocapsid-coated wells to obtain the nucleocapsid Ab signal. Each assay plate for the nucleocapsid Ab ELISA included three reference sera, which served as a control to accommodate plate variation. Similarly, a serially diluted monoclonal antibody titer was incorporated in the RBD Ab ELISA. The optical density (OD) thresholds for seropositivity in the RBD (≥0.50) and Nucleocapsid (≥0.50) assays were used based on reference panel performance. Participants were categorized as having prior SARS-CoV-2 infection if both anti-spike total Ig RBD and anti-nucleocapsid IgG thresholds for seropositivity were met.

Approach to determining prior hospitalization at time of SARS-CoV-2 positive serology

Prior hospitalization for COVID-19 or respiratory infection was based on self-report from questionnaires administered at MWCCS visits. Specifically, the question asked: “Have you ever been hospitalized because you had COVID-19 or because you had difficulty breathing or a respiratory infection.” Hospitalizations were limited to ones that occurred after January 1, 2020. A participant was considered to have had a prior hospitalization for COVID-19 or respiratory infection if they reported any hospitalization date prior to their first positive SARS-CoV-2 serology and after their initial PFT visit.

Approach to determining prior vaccination at time of SARS-CoV-2 positive serology

Prior vaccination for SARS-CoV-2 infection was based on questionnaires administered at MWCCS visits. A participant was considered to have had a prior vaccination for SARS-CoV-2 if they reported receipt of at least one dose of any SARS-CoV-2 vaccine prior to their positive SARS-CoV-2 serology.

Approach to select PFT testing

Some participants had multiple PFT visit dates that could qualify as their pre-SARS-CoV-2 PFT, as well as multiple PFT visit dates that could qualify as their post-SARS-CoV-2 PFT. To select the spirometry visit used in the analysis, we ranked the qualifying visit dates by the reader quality score of the post-BD FEV1 test. We chose the visit date that had the highest ranked post-BD FEV1 quality score and used all spirometry tests from that date. In the case of ranked ties for post-BD FEV1, we chose the date with the highest ranked post-BD FVC within the tie. If ties persisted, we chose the highest ranked pre-BD FEV1 followed by the highest ranked pre-BD FVC. For remaining ties, we chose the PFT date closest to the date of SARS-CoV-2 serology. We applied a similar algorithm when a participant had multiple visits with DLCO measurements pre- and post-SARS-CoV-2 positive serology. We ranked the visit dates by DLCO reader quality score and chose the dates with the highest ranked quality. Thus, participants could have different dates for their DLCO measurement and their FEV1/FVC measurements.

Supplementary Tables

Supplementary Table S1. Missingness in pre-positive SARS-COV-2 serology PFTs by HIV serostatus and source cohort

|  | Men  Missing (%) | |  | Women  Missing (%) | |
| --- | --- | --- | --- | --- | --- |
|  | MWH  N = 108 | MWoH  N = 96 | | WWH  N = 404 | WWoH  N = 170 |
| Pre-BD |  |  | |  |  |
| FEV1 | 6 (6%) | 8 (7%) | | 10 (6%) | 20 (5%) |
| FVC | 7 (7%) | 12 (11%) | | 24 (14%) | 54 (13%) |
| Post-BD |  |  | |  |  |
| FEV1 | 4 (4%) | 6 (6%) | | 22 (13%) | 38 (9%) |
| FVC | 7 (7%) | 9 (8%) | | 31 (18%) | 58 (14%) |
| Adjusted^a^ DLCO % predicted | 12 (12%) | 17 (16%) | | 110 (65%) | 273 (68%) |

BD: bronchodilator; DLCO: diffusing capacity of the lung for carbon monoxide; FVC: forced vital capacity; FEV_1_: forced expiratory volume in one second; MWH: men with HIV; MWoH: men without HIV; PFT: pulmonary function test; WWH: women with HIV; WWoH: women without HIV.

^a^ Adjusted for hemoglobin and carboxyhemoglobin

Supplementary Table S2. St. George’s Respiratory Questionnaire results pre- and post- first positive SARS-COV-2 serology

|  |  | | | Men | |  | Women | |
| --- | --- | --- | --- | --- | --- | --- | --- | --- |
| Domain |  | | | MWH  N = 108 | MWoH  N = 96 |  | WWH  N = 404 | WWoH  N = 170 |
| Pre-SARS-CoV-2 | |  | |  |  |  |  |  |
| Symptoms | Mean (SD) | | | 17.9 (20.7) | 12.8 (14.0) |  | 17.1 (19.8) | 17.6 (18.7) |
|  | Missing | | | 2 | 3 |  | 2 | 1 |
| Activity | Mean (SD) | | | 13.8 (20.0) | 10.5 (15.7) |  | 30.2 (26.9) | 28.6 (25.6) |
|  | Missing | | | 2 | 3 |  | 3 | 2 |
| Impacts | Mean (SD) | | | 5.2 (9.1) | 2.7 (7.4) |  | 8.1 (13.3) | 9.0 (13.3) |
|  | Missing | | | 2 | 3 |  | 2 | 1 |
| Total | Mean (SD) | | | 9.8 (12.7) | 6.5 (9.7) |  | 16.8 (16.6) | 16.8 (16.0) |
|  | Missing | | | 2 | 3 |  | 3 | 2 |
|  |  | | |  |  |  |  |  |
| Post-SARS-CoV-2 | | |  |  |  |  |  |  |
| Symptoms | Mean (SD) | | | 14.8 (16.8) | 13.6 (16.6) |  | 15.2 (19.9) | 16.5 (20.3) |
|  | Missing | | | 6 | 1 |  | 5 | 1 |
| Activity | Mean (SD) | | | 15.4 (22.1) | 10.9 (16.7) |  | 26.7 (27.2) | 30.8 (28.7) |
|  | Missing | | | 6 | 1 |  | 8 | 2 |
| Impacts | Mean (SD) | | | 4.3 (8.8) | 2.4 (6.0) |  | 7.8 (12.1) | 8.6 (13.4) |
|  | Missing | | | 6 | 1 |  | 5 | 1 |
| Total | Mean (SD) | | | 9.3 (12.3) | 6.7 (9.4) |  | 14.8 (15.8) | 16.7 (17.2) |
|  | Missing | | | 6 | 1 |  | 8 | 2 |

MWH: men with HIV; MWoH: men without HIV; SD: standard deviation; WWH: women with HIV; WWoH: women without HI

Supplementary Table S3. Adjusted difference in annualized changes in pulmonary function, measured by FEV_1_, FVC, and DLCO, before and after SARS-CoV-2 infection, by HIV serostatus

|  | MWH Change - MWoH Change (95% CI) | | | | | |  | | | WWH Change - WWoH Change (95% CI) | | | |
| --- | --- | --- | --- | --- | --- | --- | --- | --- | --- | --- | --- | --- | --- |
|  | Unadjusted | | Model 1^a^ | | Model 2^b^ | Model 3^c^ | |  | Unadjusted | | Model 1^a^ | Model 2^b^ | Model 3^c^ |
| FEV1 ml/year |  | |  | |  |  | |  |  | |  |  |  |
| Pre-BD | -4.8 (-20.2, 10.5) | | -4.8 (-20.3, 10.6) | | -1.8 (-17.7, 14.0) | -1.8 (-17.7, 14.1) | |  | -1.1(-11.0, 8.8) | | -3.9 (-13.5, 5.8) | -1.5 (-11.6, 8.6) | -4.7 (-14.5, 5.2) |
| Post-BD | -10.5 (-30.7, 9.7) | | -9.8 (-30.0, 10.4) | | -3.7 (-24.1, 16.8) | -3.0 (-23.4, 17.5) | |  | -5.0 (-18.2, 8.2) | | -8.9 (-21.6, 3.8) | -6.1 (-19.6, 7.3) | -11.0 (-23.9, 2.0) |
| FEV1 % predicted/year | |  | |  |  |  | |  |  | |  |  |  |
| Pre-BD | -0.2 (-0.7, 0.3) | | -0.2 (-0.7, 0.3) | | -0.1 (-0.6, 0.4) | -0.1 (-0.6, 0.4) | |  | -0.0 (-0.4, 0.4) | | -0.1 (-0.5, 0.3) | -0.0 (-0.4, 0.4) | -0.1 (-0.5, 0.3) |
| Post-BD | -0.3 (-0.9, 0.3) | | -0.4 (-0.9, 0.2) | | -0.1 (-0.7, 0.5) | -0.2 (-0.8, 0.4) | |  | -0.2 (-0.7, 0.3) | | -0.2 (-0.7, 0.3) | -0.2 (-0.7, 0.3) | -0.3 (-0.8, 0.2) |
| FVC mL/year |  | |  | |  |  | |  |  | |  |  |  |
| Pre-BD | -9.3 (-27.0, 8.3) | | -9.2 (-26.9, 8.5) | | -9.4 (-27.5, 8.8) | -9.2 (-27.4, 9.0) | |  | -1.1 (-12.9, 10.8) | | -5.5 (-17.1, 6.2) | 0.0 (-12.0, 12.0) | -4.4 (-16.3, 7.4) |
| Post-BD | 2.5 (-15.5, 20.5) | | 2.4 (-15.5, 20.3) | | 6.1 (-12.5, 24.6) | 6.1 (-12.4, 24.6) | |  | -4.3 (-17.3, 8.8) | | -8.6 (-21.4, 4.1) | -4.2 (-17.4, 9.1) | -9.0 (-22.0, 3.9) |
| FVC % predicted/year |  | |  | |  |  | |  |  | |  |  |  |
| Pre-BD | -0.3 (-0.7, 0.2) | | -0.3 (-0.7, 0.1) | | -0.2 (-0.7, 0.2) | -0.3 (-0.7, 0.2) | |  | -0.0 (-0.4, 0.4) | | -0.1 (-0.5, 0.3) | 0.0 (-0.4, 0.4) | -0.1 (-0.4, 0.3) |
| Post-BD | 0.0 (-0.4, 0.5) | | -0.0 (-0.5, 0.4) | | 0.1 (-0.3, 0.6) | 0.1 (-0.4, 0.5) | |  | -0.1 (-0.5, 0.3) | | -0.2 (-0.6, 0.2) | -0.1 (-0.5, 0.3) | -0.2 (-0.6, 0.2) |
| DLCO ^d^ % predicted/year | -0.2 (-0.9, 0.5) | | -0.3 (-1.0, 0.4) | | -0.1 (-0.8, 0.6) | -0.2 (-0.9, 0.5) | |  | 0.1 (-1.2, 1.4) | | -0.0 (-1.3, 1.2) | 0.2 (-1.1, 1.5) | 0.0 (-1.3, 1.3) |

BD: bronchodilator; CI: confidence interval; DLCO: diffusing capacity of the lung for carbon monoxide;

FEV1: forced expiratory volume in one second; FVC: forced vital capacity; MWH: men with HIV; MWoH: men without HIV; PFT: pulmonary function test; WWH: women with HIV; WWoH: women without HIV.

^a^Model 1 adjusted for respective baseline lung function measure; ^b^Model 2 adjusted for current smoking status; ^c^Model 3 adjusted for respective baseline lung function measure and current smoking status; ^d^Adjusted for hemoglobin and carboxyhemoglobin

Supplementary Table S4. Cohort characteristics stratified by decline in % predicted FEV1

|  | Men | |  | | Women | |
| --- | --- | --- | --- | --- | --- | --- |
|  | 10% with largest % predicted FEV1 decliner  N = 18 | Remaining 90%  N = 157 | | 10% with largest % predicted FEV1 decliner  N = 46 | | Remaining 90%  N = 355 |
| Demographic Characteristics |  |  | |  | |  |
| Age, median (Q1, Q3) | 58 (54, 66) | 61 (52, 67) | | 57 (48, 64) | | 53 (45, 59) |
| Race and ethnicity, n (%) |  |  | |  | |  |
| Black, non-Hispanic | 9 (50.0) | 45 (28.7) | | 31 (72.1) | | 313 (81.1) |
| White, non-Hispanic | 8 (44.4) | 88 (56.1) | | 2 (4.7) | | 19 (4.9) |
| Another race, non-Hispanic | 0 (0.0) | 4 (2.5) | | 2 (4.7) | | 12 (3.1) |
| Any race, Hispanic | 1 (5.6) | 20 (12.7) | | 8 (18.6) | | 42 (10.9) |
| Annual income ≤ $18,000, n (%) | 5 (35.7) | 26 (17.4) | | 28 (66.7) | | 170 (48.2) |
| Region, n (%) |  |  | |  | |  |
| West | 2 (11.1) | 39 (24.8) | | 2 (4.7) | | 16 (4.1) |
| Northeast | 0 (0.0) | 1 (0.6) | | 10 (23.3) | | 145 (37.6) |
| Mid-Atlantic | 7 (38.9) | 43 (27.4) | | 9 (20.9) | | 23 (6.0) |
| South | 9 (50.0) | 74 (47.1) | | 21 (48.8) | | 188 (48.7) |
| Midwest |  |  | | 1 (2.3) | | 14 (3.6) |
| Clinical Characteristics^a^ |  |  | |  | |  |
| SARS-CoV-2 vaccination, n (%) | 17 (100.0) | 134 (89.9) | | 30 (69.8) | | 270 (70.9) |
| Missing | 1 | 8 | | 0 | | 5 |
| Prior hospitalization, n (%) | 2 (11.8) | 11 (7.3) | | 5 (11.9) | | 20 (5.3) |
| Missing | 1 | 7 | | 1 | | 8 |
| Hypertension, n (%) | 11 (61.1) | 85 (54.1) | | 28 (65.1) | | 243 (63.0) |
| Diabetes, n (%) | 4 (22.2) | 39 (24.8) | | 14 (32.6) | | 103 (26.7) |
| Myocardial infarction, n (%) | 0 (0.0) | 2 (1.3) | | 1 (2.3) | | 9 (2.4) |
| COPD (ever), n (%) | 1 (5.9) | 6 (4.0) | | 10 (23.3) | | 29 (7.7) |
| Asthma (ever), n (%) | 1 (5.9) | 12 (7.9) | | 12 (27.9) | | 83 (22.1) |
| Kidney disease/renal failure, n (%) | 2 (11.8) | 6 (4.0) | | 2 (4.7) | | 8 (2.1) |
| Race-free eGFR<60, n (%) | 1 (5.6) | 20 (12.7) | | 9 (20.9) | | 55 (14.2) |
| PWH, n (%) | 13 (72.2) | 80 (51.0) | | 33 (76.7) | | 275 (71.2) |
| HIV viral load, n (%) |  |  | |  | |  |
| Undetectable: no signal | 7 (53.8) | 44 (55.0) | | 17 (51.5) | | 171 (62.2) |
| Undetectable: under lower limit | 3 (23.1) | 11 (13.8) | | 4 (12.1) | | 40 (14.5) |
| Detectable | 3 (23.1) | 25 (31.2) | | 12 (36.4) | | 64 (23.3) |
| CD4, cells/mm^3^, median (Q1, Q3) | 762 (637, 906) | 787 (562, 977) | | 748 (627, 1110) | | 850 (636, 1125) |
| BMI, median (Q1, Q3) | 28 (24, 32) | 27 (24, 30) | | 35 (28, 38) | | 32 (27, 39) |
| Underweight (<18.5) | 1 (5.6) | 1 (0.6) | | 0 (0.0) | | 2 (0.5) |
| Healthy weight (18.5 to <25) | 5 (27.8) | 48 (30.6) | | 5 (11.6) | | 48 (12.4) |
| Overweight (25 to <30) | 4 (22.2) | 70 (44.6) | | 9 (20.9) | | 92 (23.8) |
| Obese (30+) | 8 (44.4) | 38 (24.2) | | 29 (67.4) | | 244 (63.2) |
| Hepatitis C status, n (%) | 1 (5.6) | 3 (1.9) | | 0 (0.0) | | 8 (2.1) |
| Hepatitis B status, n (%) | 2 (11.1) | 5 (3.2) | | 2 (4.7) | | 3 (0.8) |
| Behavioral Characteristics |  |  | |  | |  |
| Current tobacco smoking, n (%) | 6 (33.3) | 19 (12.1) | | 12 (27.9) | | 386 (25.4) |
| Ever tobacco smoking, n (%) | 12 (66.7) | 95 (60.5) | | 28 (65.1) | | 218 (56.5) |
| Pack-years, median (Q1, Q3) | 19 ( 6, 45) | 11 ( 4, 25) | | 8 ( 3, 13) | | 7 ( 3, 16) |

COPD: chronic obstructive pulmonary disease; eGFR: estimated glomerular filtration rate; Q1: first quartile; Q3: third quartile; MWH: men with HIV; MWoH: men without HIV; WWH: women with HIV; WWoH: women without HIV.

^a^See methods and supplementary information for comorbidity definitions

Supplementary Table S5. Percent of participant with improvement (decrease) or worsening (increase) of at least 4 percentage points in St. George’s Respiratory Questionnaire domains

|  |  | Men  Percentage (95% CI) | |  | | Women  Percentage (95% CI) | |
| --- | --- | --- | --- | --- | --- | --- | --- |
| Domain | Change ≥ 4 points | MWH  N = 108^a^ | MWoH  N = 96^b^ | | WWH  N = 404^c^ | | WWoH  N = 170^d^ |
| Symptoms | Worsening | 31 (22-40) | 34 (24-43) | | 29 (24-33) | | 36 (29-44) |
|  | Improvement | 33 (24-42) | 40 (30-50) | | 39 (34-44) | | 38 (31-45) |
|  | No change | 36 (27-45) | 26 (17-35) | | 32 (28-37) | | 26 (19-32) |
|  |  |  |  | |  | |  |
| Activity | Worsening | 35 (26-44) | 29 (20-39) | | 36 (31-40) | | 43 (35-50) |
|  | Improvement | 30 (21-39) | 32 (22-41) | | 45 (41-50) | | 39 (32-46) |
|  | No change | 35 (26-44) | 39 (29-49) | | 19 (15-23) | | 19 (13-24) |
|  |  |  |  | |  | |  |
| Impacts | Worsening | 16 (9-23) | 12 (5-19) | | 25 (20-29) | | 23 (17-30) |
|  | Improvement | 17 (10-24) | 14 (7-21) | | 27 (23-32) | | 34 (27-41) |
|  | No change | 67 (58-76) | 74 (65-83) | | 48 (43-53) | | 43 (35-50) |
|  |  |  |  | |  | |  |
| Total | Worsening | 27 (18-36) | 20 (11-28) | | 28 (23-32) | | 29 (22-36) |
|  | Improvement | 22 (14-30) | 23 (14-31) | | 38 (33-43) | | 35 (28-42) |
|  | No change | 51 (41-61) | 58 (48-68) | | 34 (30-39) | | 37 (29-44) |

MWH: men with HIV; MWoH: men without HIV; WWH: women with HIV; WWoH: women without HIV.

^a^ 8 (7%) missing.

^b^ 4 (4%) missing.

^c^ 7 (2%) missing Symptoms and Impact domains, 10 (2%) missing Activity domain

^d^ 2 (1%) missing Symptoms and Impact domains, 3 (2%) missing Activity domain

**SUPPLEMENTARY FIGURES**

Figure S1. CONSORT diagram (MWH: men with HIV; MWoH: men without HIV; PFT: pulmonary function test; WWH: women with HIV; WWoH: women without HIV)


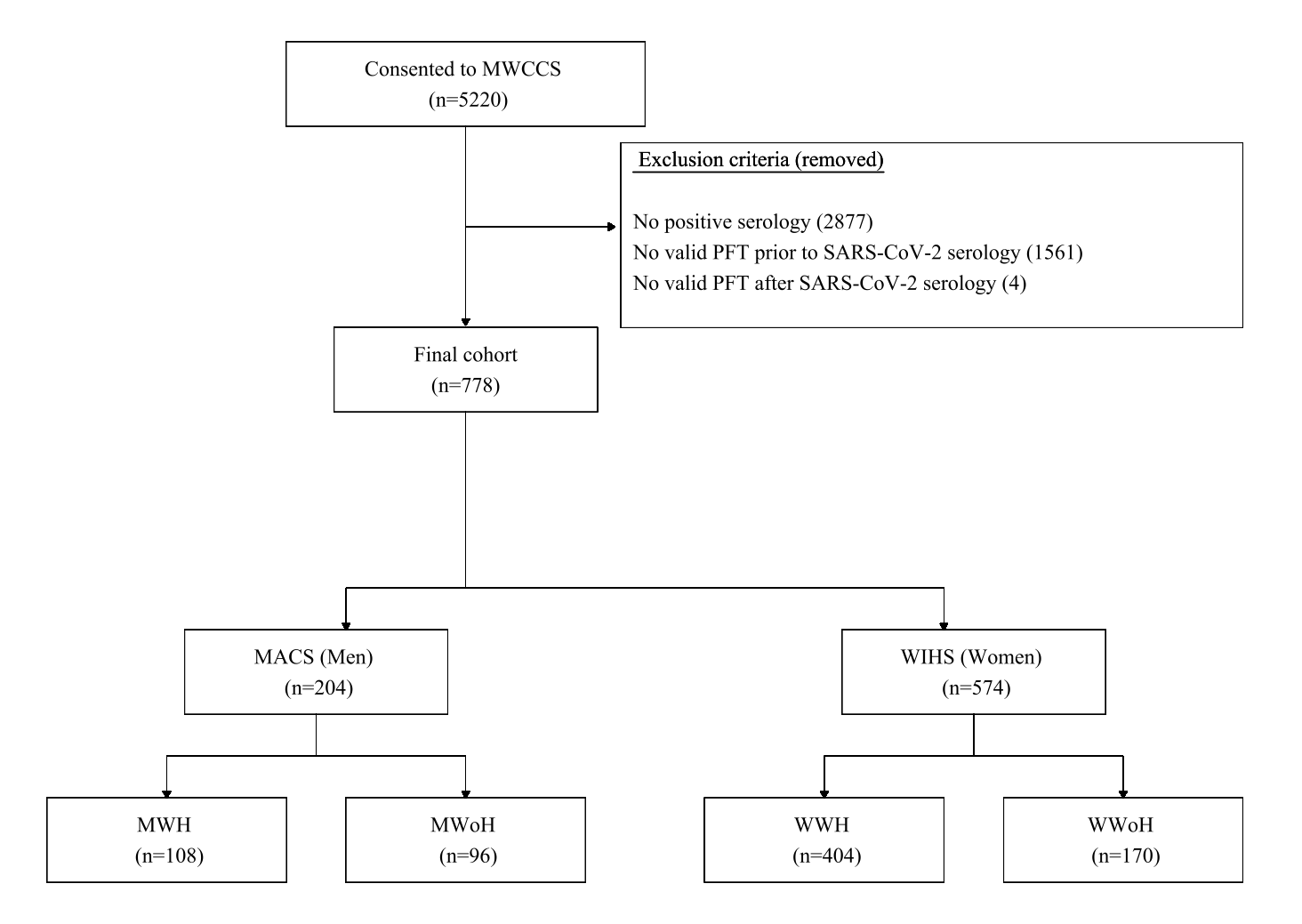


FIGURE S2. Difference between HIV serostatus groups in change of post-BD FEV1 (ml/year) between pulmonary function assessments pre- and post-SARS-CoV-2 positive serology among men (MWH: men with HIV; MWoH: men without HIV)


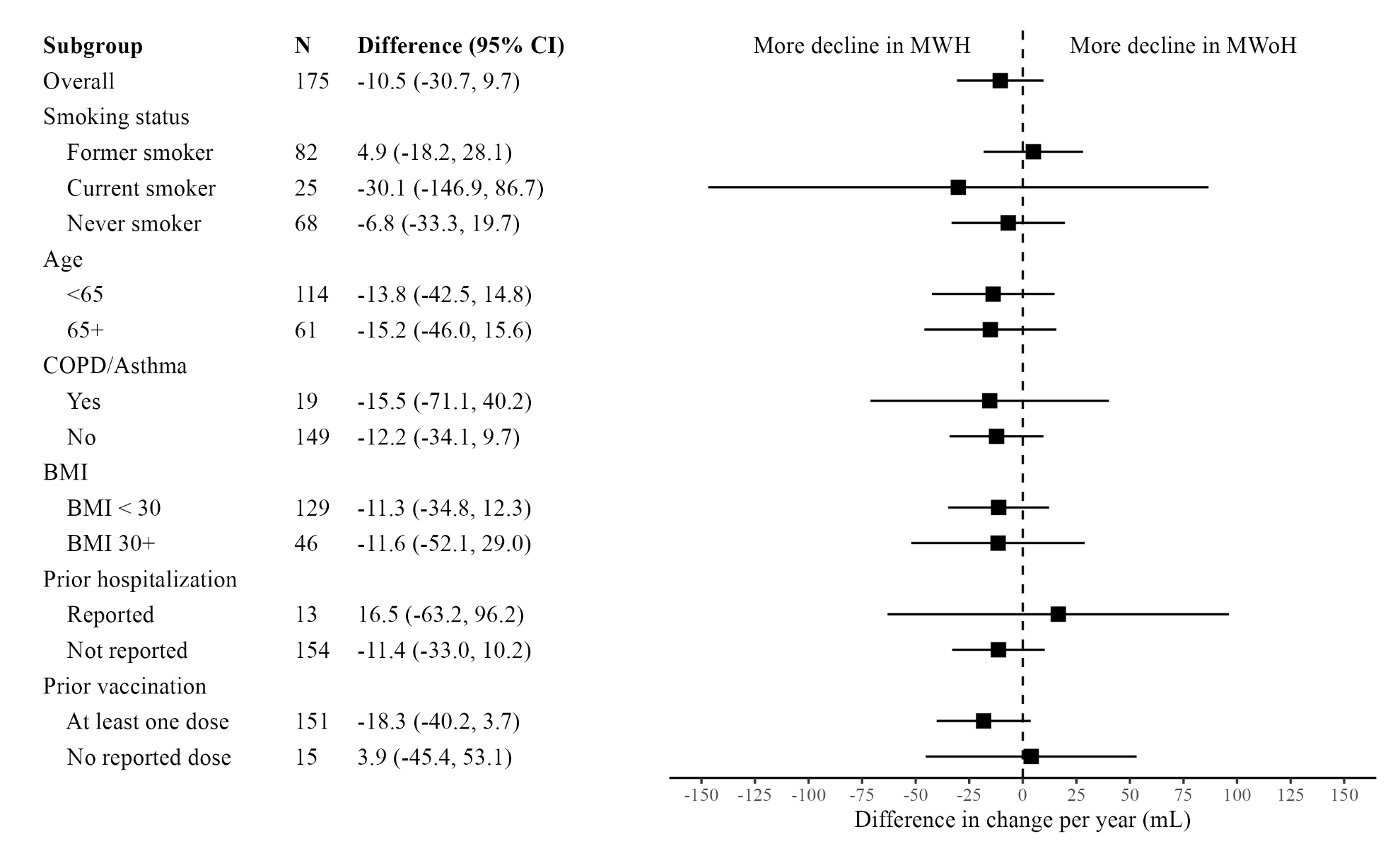


FIGURE S3. Difference between HIV serostatus groups in change of post-BD FEV1 (ml/year) between pulmonary function assessments pre- and post-SARS-CoV-2 positive serology among women (WWH: women with HIV; WWoH: women without HIV)


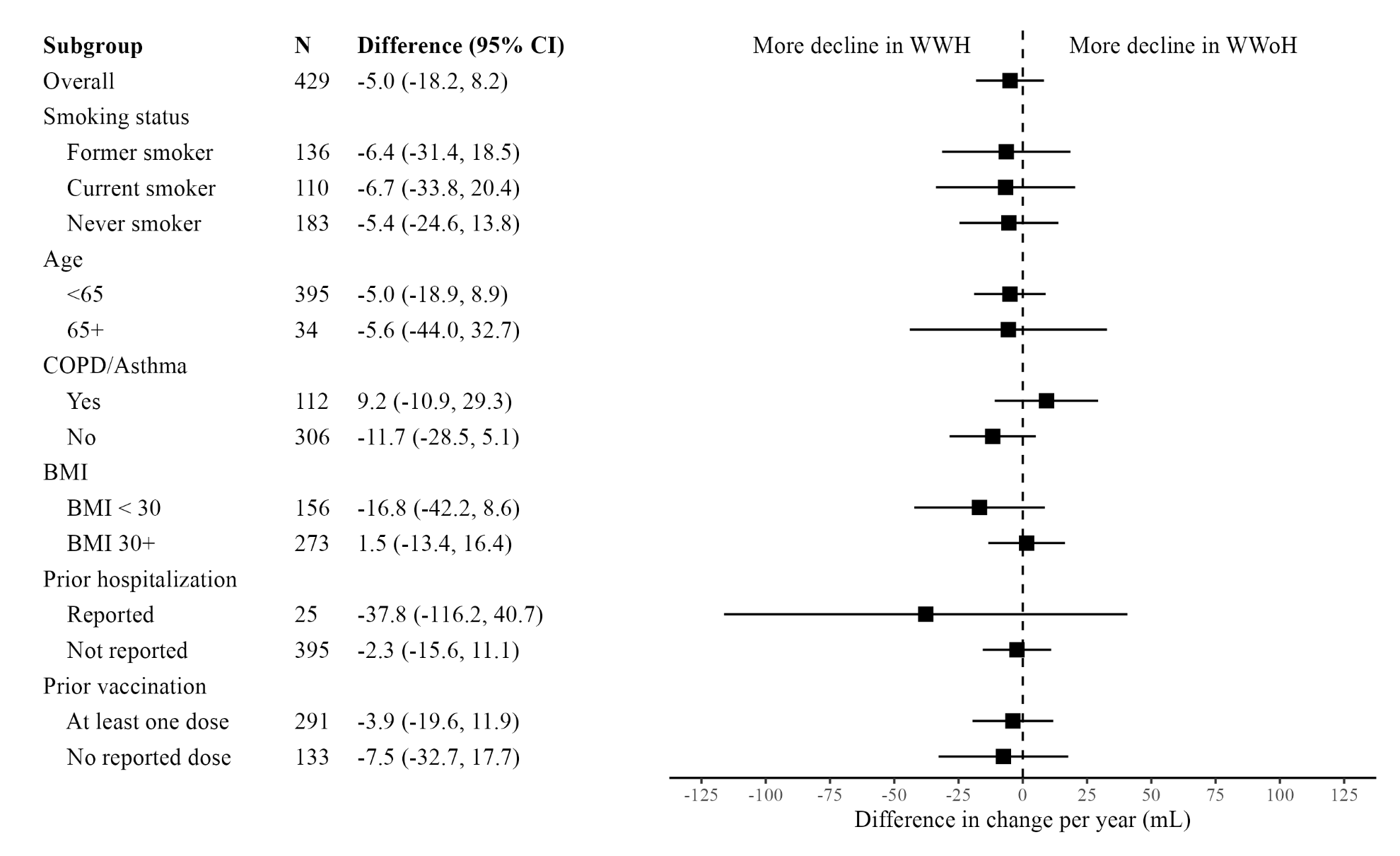


FIGURE S4. Difference between HIV serostatus groups in change of post-BD FVC (ml/year) between pulmonary function assessments pre- and post-SARS-CoV-2 positive serology among men (MWH: men with HIV; MWoH: men without HIV)


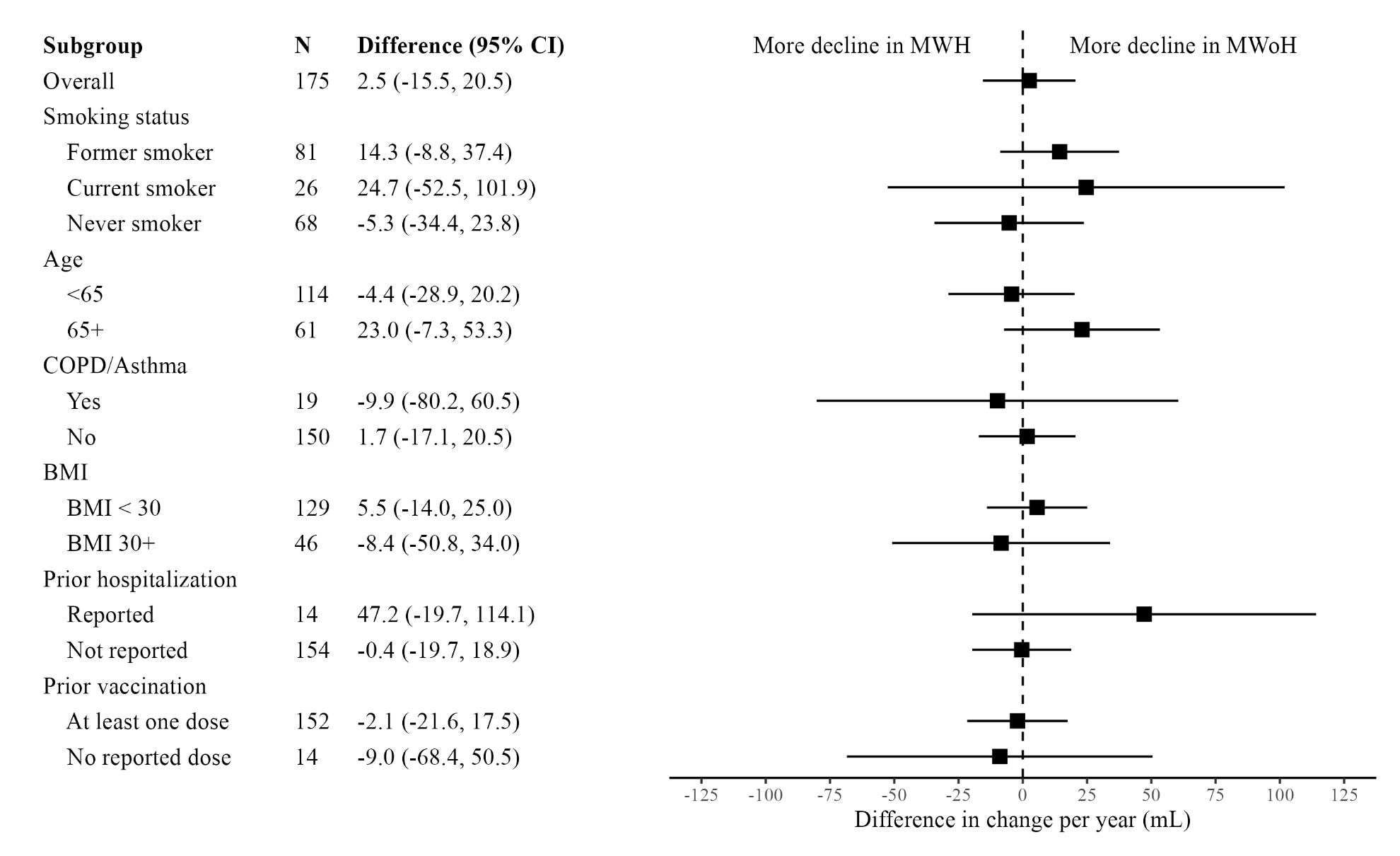


FIGURE S5. Difference between HIV serostatus groups in change of post-BD FVC (ml/year) between pulmonary function assessments pre- and post-SARS-CoV-2 positive serology among women (WWH: women with HIV; WWoH: women without HIV)


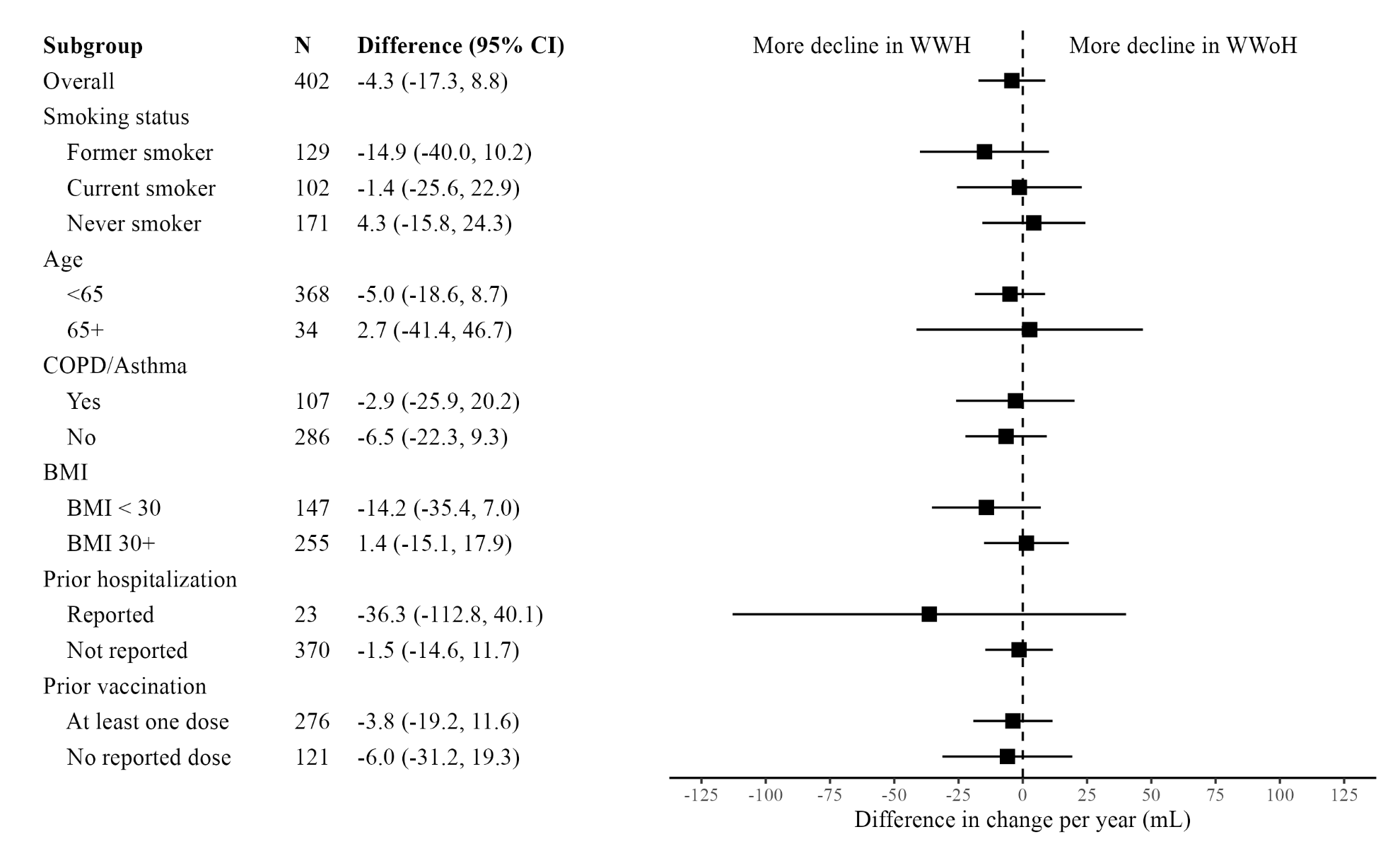


FIGURE S6. Difference between HIV serostatus groups in change of DLCO adjusted for hemoglobin and carboxyhemoglobin (% predicted per year) between pulmonary function assessments pre- and post-SARS-CoV-2 positive serology among men (MWH: men with HIV; MWoH: men without HIV


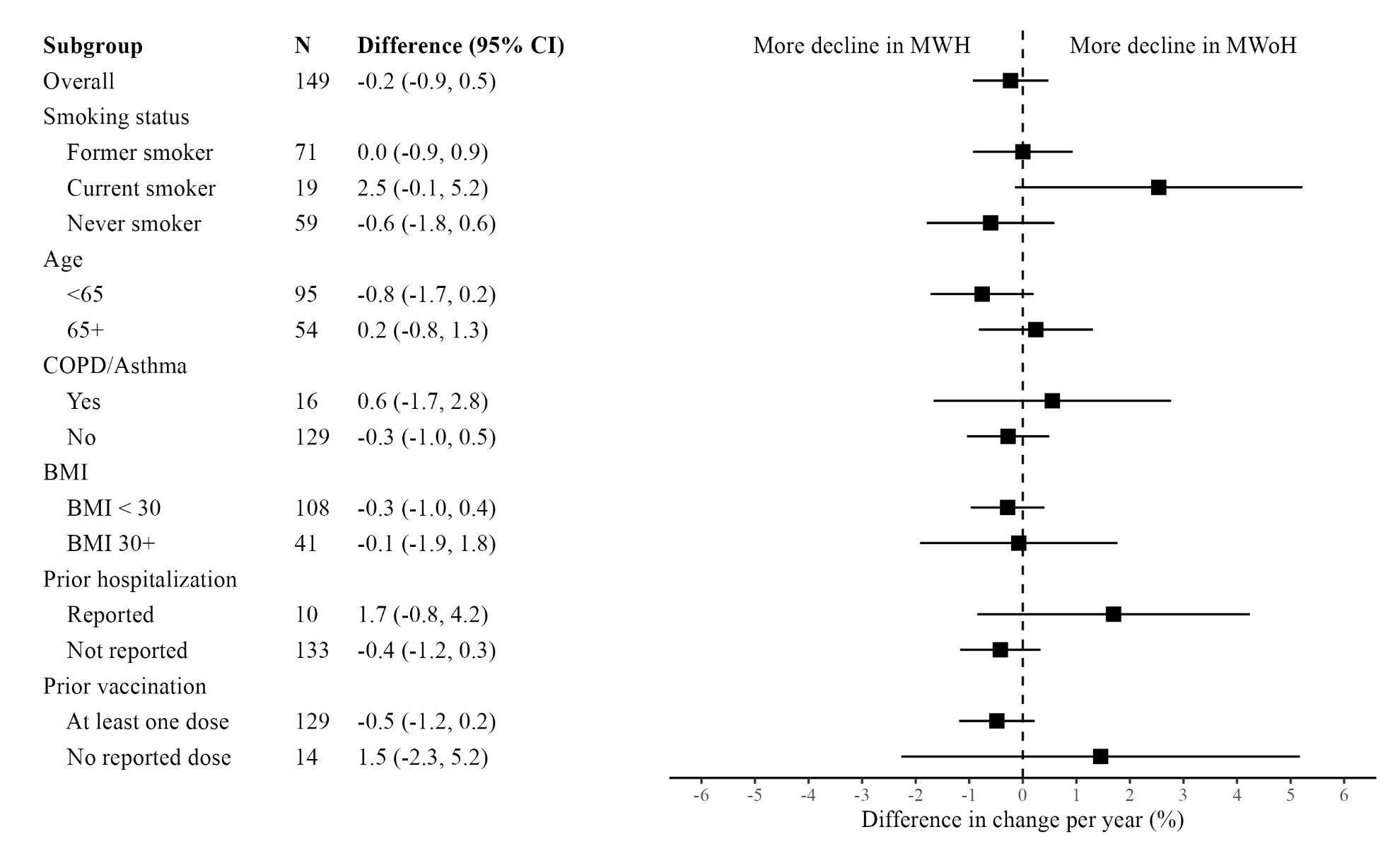


FIGURE S7. Difference between HIV serostatus groups in change of DLCO adjusted for hemoglobin and carboxyhemoglobin (% predicted per year) between pulmonary function assessments pre- and post-SARS-CoV-2 positive serology among women (WWH: women with HIV; WWoH: women without HIV)


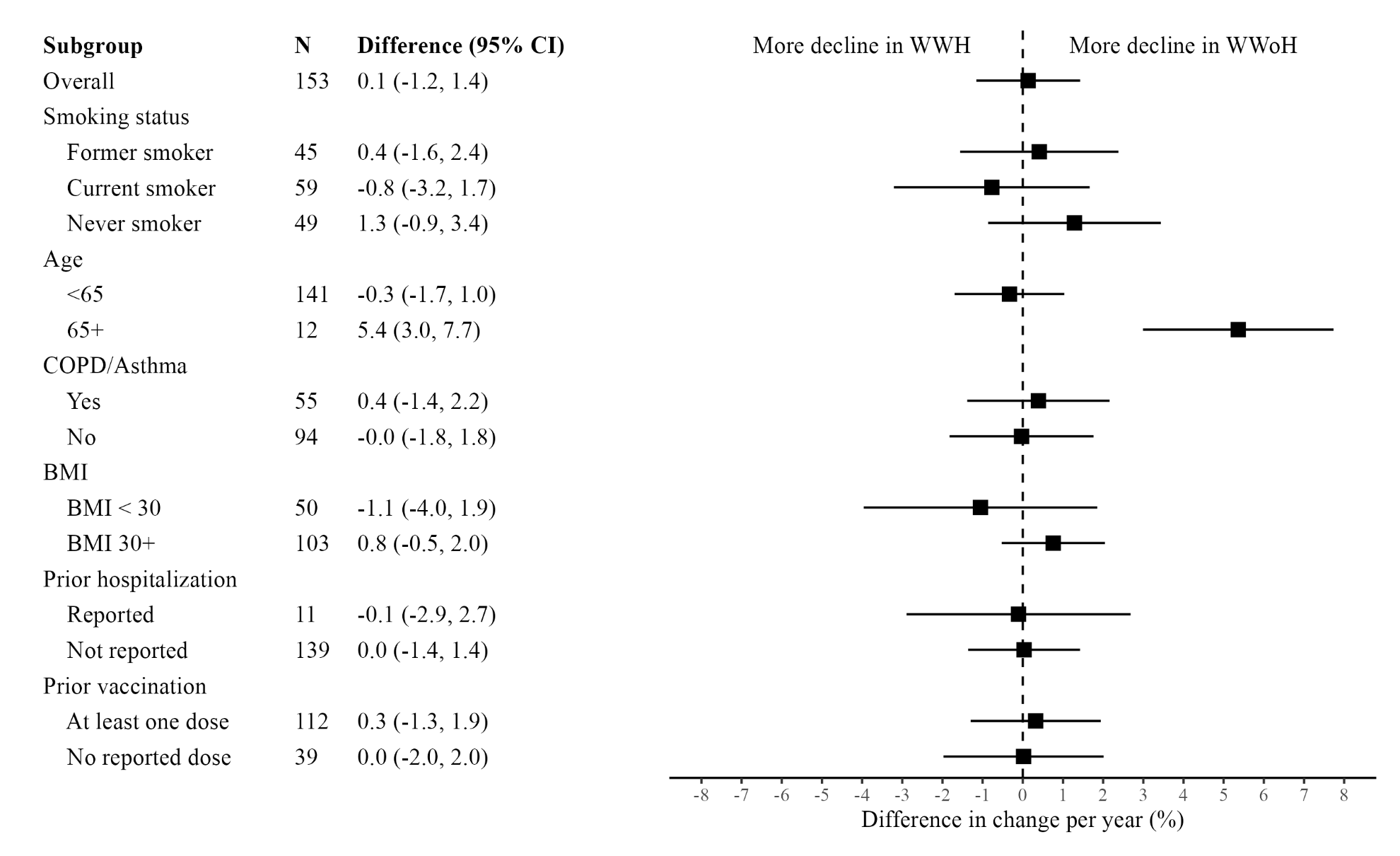


FIGURE S8. Unadjusted and adjusted difference between HIV serostatus groups in change of post-BD FEV1 (ml/year) between pulmonary function assessments pre- and post-SARS-CoV-2 positive serology (PWH: people with HIV, PWoH: people without HIV)


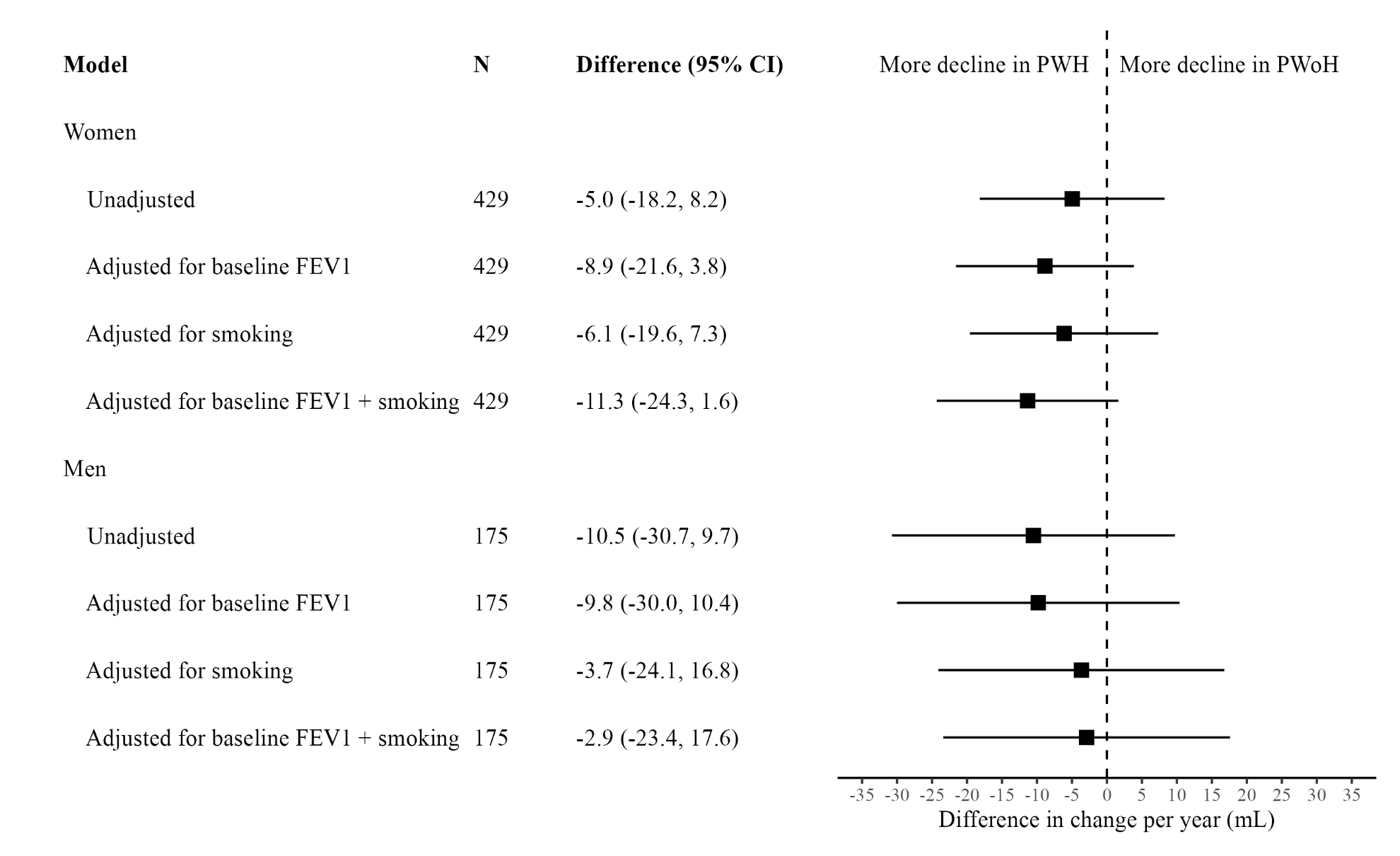


FIGURE S9. Unadjusted and adjusted difference between HIV serostatus groups in change of post-BD FVC (ml/year) between pulmonary function assessments pre- and post-SARS-CoV-2 positive serology (PWH: people with HIV, PWoH: people without HIV)


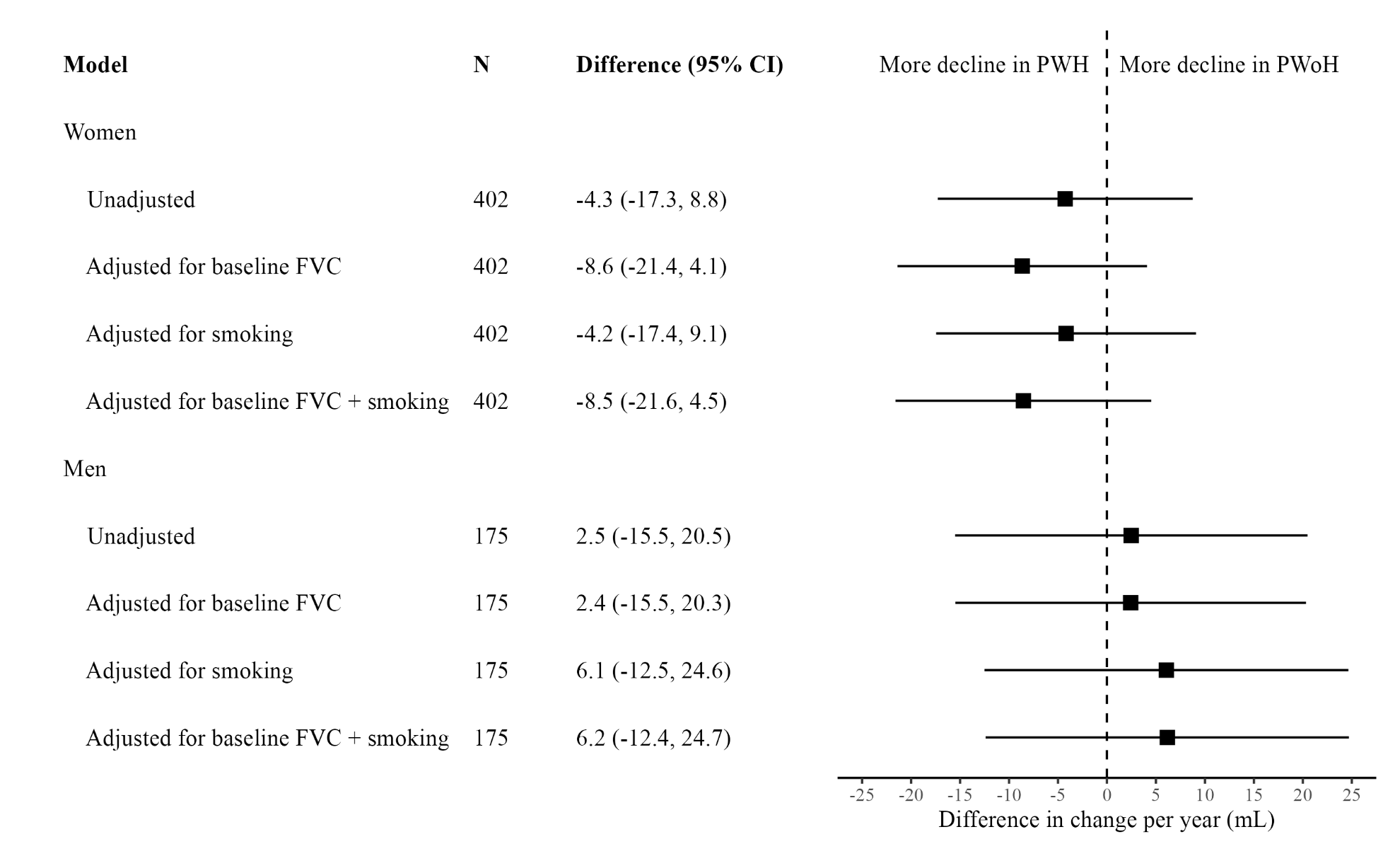


FIGURE S10. Unadjusted and adjusted difference between HIV serostatus groups in change of DLCO adjusted for hemoglobin and carboxyhemoglobin (% predicted per year) between pulmonary function assessments pre- and post-SARS-CoV-2 positive serology (PWH: people with HIV, PWoH: people without HIV)


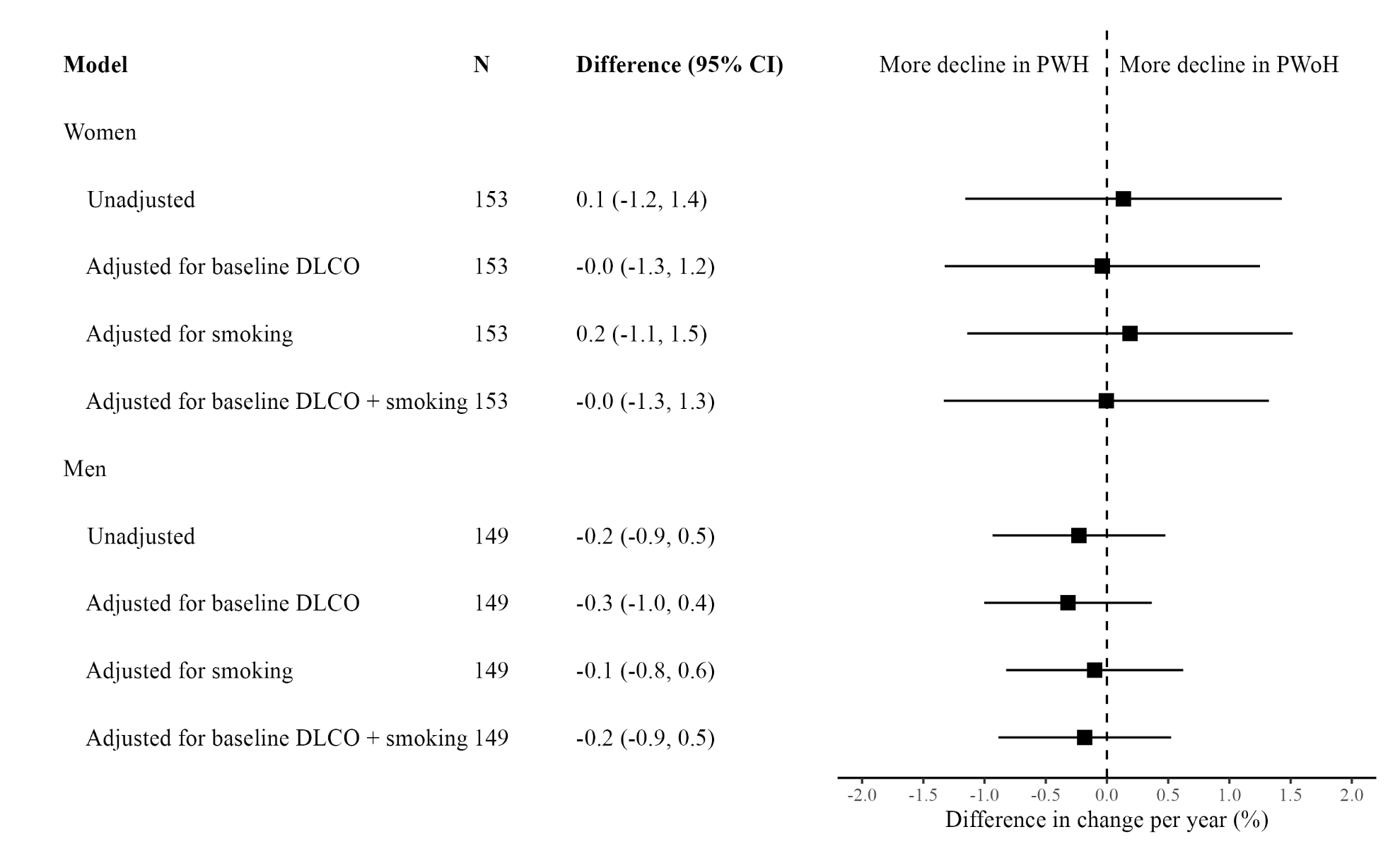


Supplementary References

1. Narowski TM, Raphel K, Adams LE, Huang J, Vielot NA, Jadi R, de Silva AM, Baric RS, Lafleur JE, Premkumar L. SARS-CoV-2 mRNA vaccine induces robust specific and cross-reactive IgG and unequal neutralizing antibodies in naive and previously infected people. *Cell Rep* 2022; 38: 110336.

2. Premkumar L, Segovia-Chumbez B, Jadi R, Martinez DR, Raut R, Markmann A, Cornaby C, Bartelt L, Weiss S, Park Y, Edwards CE, Weimer E, Scherer EM, Rouphael N, Edupuganti S, Weiskopf D, Tse LV, Hou YJ, Margolis D, Sette A, Collins MH, Schmitz J, Baric RS, de Silva AM. The receptor binding domain of the viral spike protein is an immunodominant and highly specific target of antibodies in SARS-CoV-2 patients. *Sci Immunol* 2020; 5.
